# Supplementary material for: Multiomics approaches disclose very-early molecular and cellular switches during insect-venom allergen-specific immunotherapy: an observational study
Source: Nat Commun. 2024 Nov 26;15:10266. doi: 10.1038/s41467-024-54684-2 (PMC11599746; doi:10.1038/s41467-024-54684-2)
Supplement: Supplementary file 1 — Supplementary Information [file 41467_2024_54684_MOESM1_ESM.pdf]

Multiomics approaches disclose very-early molecular and cellular switches during insect-venom allergen-specific immunotherapy: an observational study

Supplementary Information

Dimitrii Pogorelov[1, 2, 18, #], Sebastian Felix Nepomuk Bode[1, 3, 4, #], Xin He[5, #], Javier Ramiro-Garcia[5], Fanny Hedin[6], Wim Ammerlaan[7], Maria Konstantinou[6], Christophe M. Capelle[1, 2, 20], Ni Zeng[1, 2], Aurélie Poli[1, 19], Olivia Domingues[1], Guillem Montamat[1], Oliver Hunewald[1], Séverine Ciré[1], Alexandre Baron[1], Joseph Longworth[1, 5], Agnieszka Demczuk[1, 2], Murilo Luiz Bazon[1], Ingrid Casper[8], Ludger Klimek[8], Lorie Neuberger-Castillo[7], Dominique Revets[6], Lea Guyonnet[1, 9], Sylvie Delhalle[1], Jacques Zimmer[1], Vladimir Benes[10], Françoise Codreanu-Morel[11], Christiane Lehners-Weber[11], Ilse Weets[12], Pinar Alper[5], Dirk Brenner[1, 5, 13], Jan Gutermuth[14], Coralie Guerin[1, 9], Martine Morisset[11, 15], François Hentges[11], Reinhard Schneider[5], Mohamed H. Shamji[16], Fay Betsou[7, 17], Paul Wilmes[2, 5], Enrico Glaab[5], Antonio Cosma[6], Jorge Goncalves[5], Feng Q. Hefeng[1, *], Markus Ollert[1, 13, *]

1, Department of Infection and Immunity, Luxembourg Institute of Health (LIH), Esch-sur-Alzette, Luxembourg

2, Department of Life Sciences and Medicine, Faculty of Science, Technology and Medicine, University of Luxembourg, Esch-sur-Alzette, Luxembourg

3, Department of Pediatrics and Adolescent Medicine, Ulm University Medical Center, Ulm, Germany

4, Department of General Pediatrics, Adolescent Medicine and Neonatology, Medical Centre–University of Freiburg, Faculty of Medicine, Freiburg, Germany

5, Luxembourg Centre for Systems Biomedicine, University of Luxembourg, Belvaux, Luxembourg

6, National Cytometry Platform, Luxembourg Institute of Health, Esch-sur-Alzette, Luxembourg

7, Integrated BioBank of Luxembourg, Luxembourg Institute of Health, Dudelange, Luxembourg

8, Center for Rhinology and Allergology, Wiesbaden, Germany

9, Cytometry Platform, Institut Curie; Innovative Therapies in Haemostasis, INSERM, Université de Paris, Paris, France

10, Genomics Core Facility, European Molecular Biology Laboratory, Heidelberg, Germany

11, National Unit of Immunology-Allergology, Centre Hospitalier de Luxembourg, Luxembourg, Luxembourg

12, Department of Clinical Biology/ Research Group Experimental Pharmacology, Vrije Universiteit Brussel, Universitair Ziekenhuis Brussel, Brussels, Belgium

13, Department of Dermatology and Allergy Center, Odense Research Center for Anaphylaxis, University of Southern Denmark, Odense, Denmark

14, Department of Dermatology, Vrije Universiteit Brussel, Universitair Ziekenhuis Brussel, Brussels, Belgium

15, Allergy Unit, Angers University Hospital, Angers, France

16, Immunomodulation and Tolerance Group, Allergy and Clinical Immunology, Department of National Heart and Lung Institute, Imperial College London, London, UK

17, CRBIP, Institut Pasteur, Université Paris Cité, Paris, France

18, Present address: Center of Allergy & Environment, Technical University of Munich, Munich, Germany

19, Present address: Department of Cancer Research, Luxembourg Institute of Health, Luxembourg, Luxembourg

20, Present Address: Institute of Microbiology, ETH Zurich, Zurich, Switzerland

#, These authors contributed equally.

*, These authors jointly supervised this work. Correspondence to F.Q.H. (Feng.Hefeng@lih.lu) or M.O. (Markus.Ollert@lih.lu).

Supplementary Figures

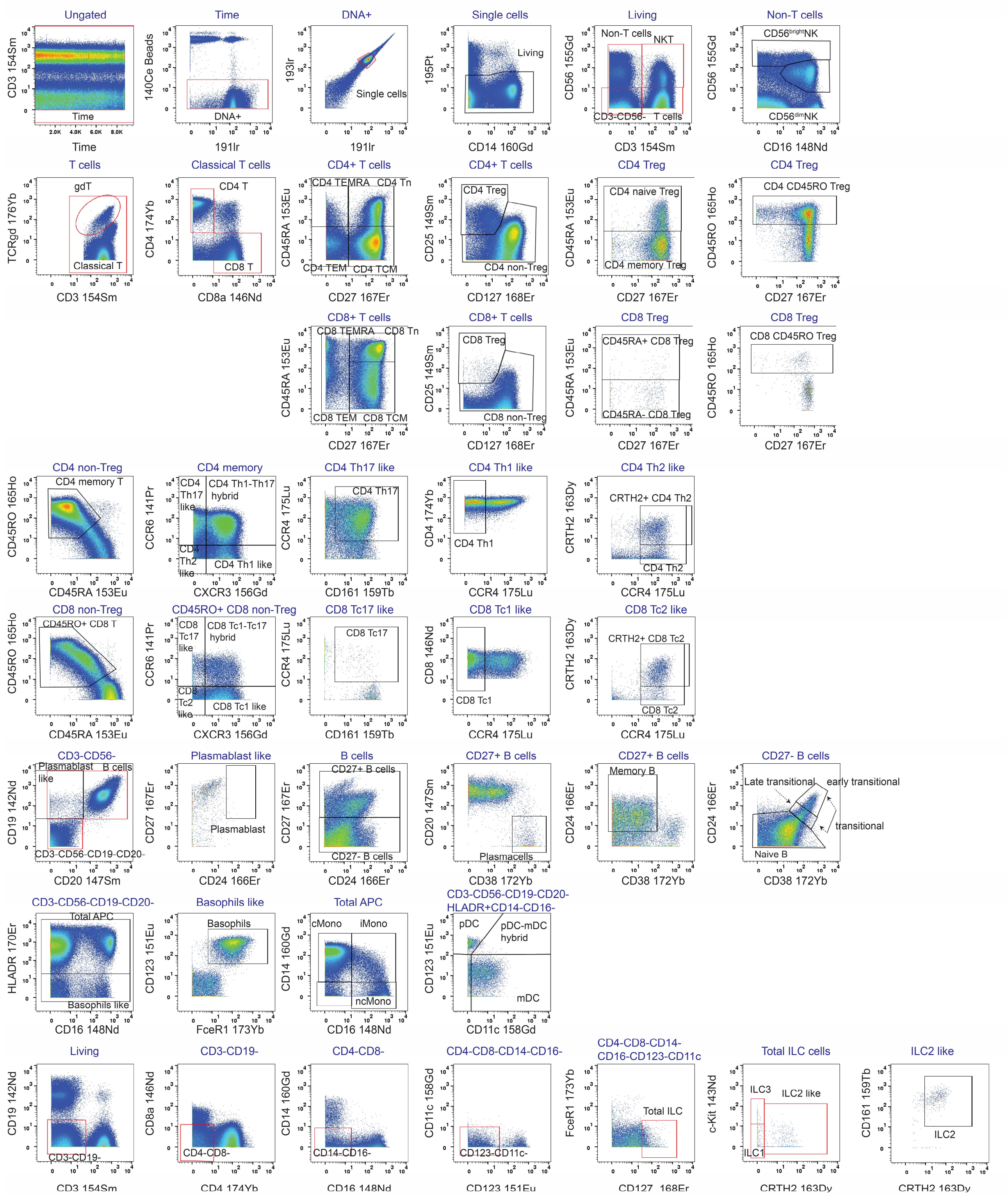

Supplementary Fig. 1. CyTOF gating strategy to identify 77 peripheral immune subsets.

Gating strategy to define various immune subsets in peripheral blood mononuclear cells (PBMCs) using manually-gated supervised mass cytometry (CyTOF) analysis. The labels and markers of major immune cell types of interests were enlarged. The corresponding parent gate was labeled in blue above each cytometry plot. APC, antigen presenting cells; $CD4^+$ Th1, type 1 helper T cells; $CD4^+$ Th2, type 2 helper T cells; $CD8^+$ Tc2, type 2 cytotoxic cells; DC, dendritic cells; γδ T cells, gamma-delta T cells; ILC1/2/3, type 1/2/3 innate lymphoid cells; cMono, classical monocytes; iMono, intermediate monocytes; ncMono, non-classical monocytes; NK, natural killer cells; NKT, natural killer T cells; non-Treg, non-regulatory T cells; mDC, myeloid DC; pDC, plasmacytoid DC; pDC-mDC, hybrid plasmacytoid and myeloid DC; $CD8^+$ Tc1, type 1 cytotoxic cells; Tc1-Tc17, hybrid type 1 and type 17 cytotoxic $CD8^+$ T cells; Th1-Th17, hybrid type 1 and type 17 $CD4^+$ T helper cells; TCM, central memory T cells; TEM, effector memory T cells; TEMRA, terminally-differentiated effector memory T cells; Tn, naïve T cells; Treg, regulatory T cells. The combinations of markers used to define the 77 subsets were provided as part of Source Data. For the full gating strategy, one could visit https://public.tableau.com/app/profile/lihpublicdata/viz/i3Dare_SYSTACT_Database/SYSTACTHome.

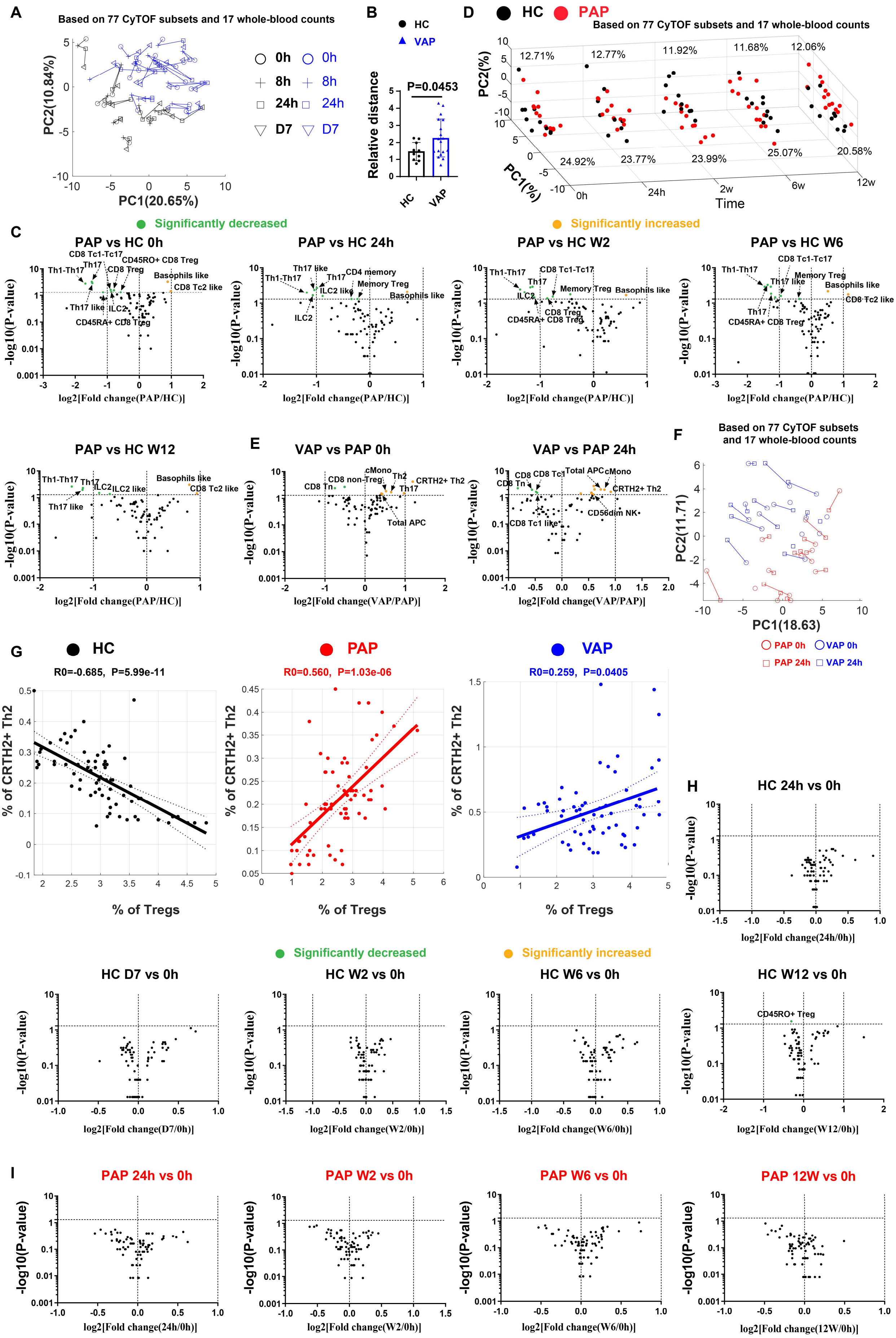
A
Based on 77 CyTOF subsets and 17 whole-blood counts
PC1(20.65%)
PC2(10.84%)
0h 0h
8h 8h
24h 24h
D7 D7
B
HC
VAP
P=0.0453
Relative distance
D
HC PAP
Based on 77 CyTOF subsets and 17 whole-blood counts
PC2(%)
PC1(%)
Time
0h 24h 2w 6w 12w
12.71% 12.77% 11.92% 11.68% 12.06%
24.92% 23.77% 23.99% 25.07% 20.58%
C
Significantly decreased
Significantly increased
PAP vs HC 0h
PAP vs HC 24h
PAP vs HC W2
PAP vs HC W6
PAP vs HC W12
-log10(P-value)
log2[Fold change(PAP/HC)]
Th1-Th17
Th17
CD8 Tc1-Tc17
CD8 Treg
CD45RO+ CD8 Treg
Basophils like
CD8 Tc2 like
Th17 like
ILC2
CD45RA+ CD8 Treg
CD4 memory
Memory Treg
E
VAP vs PAP 0h
VAP vs PAP 24h
log2[Fold change(VAP/PAP)]
CD8 Tn
CD8 non-Treg
cMono
Th2
CRTH2+ Th2
Total APC
CD8 Tc1
CD8 Tc1 like
CD56dim NK
F
Based on 77 CyTOF subsets and 17 whole-blood counts
PC1(18.63)
PC2(11.71)
PAP 0h VAP 0h
PAP 24h VAP 24h
G
HC
PAP
VAP
R0=-0.685, P=5.99e-11
R0=0.560, P=1.03e-06
R0=0.259, P=0.0405
% of CRTH2+ Th2
% of Tregs
H
HC 24h vs 0h
HC D7 vs 0h
HC W2 vs 0h
HC W6 vs 0h
HC W12 vs 0h
CD45RO+ Treg
log2[Fold change(24h/0h)]
log2[Fold change(D7/0h)]
log2[Fold change(W2/0h)]
log2[Fold change(W6/0h)]
log2[Fold change(W12/0h)]
I
PAP 24h vs 0h
PAP W2 vs 0h
PAP W6 vs 0h
PAP 12W vs 0h
log2[Fold change(12W/0h)]

67

Supplementary Fig. 2. Extended deep immunophenotyping analysis.

A, PCA plot of the samples from VAP and HC. **B**, Average immunological response within VAP following AIT launch vs. HC among the four sampling time points. **C**, Percentages of different immune subsets in PAP vs. HC at the matched time points. The list of significantly enhanced or decreased immune subsets (p<=0.05) were highlighted. **D**, Time-slice PCA plot of the samples from PAP and HC. The analysis was performed by integrating CyTOF and whole-blood-count results. **E**, Percentages of different immune subsets in VAP vs. PAP at the two matched time points. **F**, PCA plot showing the samples from different time points of VAP and PAP. **G**, Spearman correlation between the percentages of CRTH2^{+} Th2 and of Tregs among total living singlets (HC, n=70; VAP, n=63; PAP, n=66 independent biological samples). **H, I**, Percentages of different immune subsets in HC (**H**) or PAP (**I**) at the indicated later time points vs. baseline. The significantly enhanced or decreased immune subsets (p<=0.05) were highlighted. Data represent mean± S.D. (**B**). P-value was determined by paired (**H, I**) or non-paired (**C, E**) two-tailed Mann-Whitney test or a two-tailed exact permutation distribution test (**G**). Each dot in **A, D**, **F, G** represents one sample. Each dot in **B** represents one individual. The different samples from the same individual were connected with one line (**A**, **F**). AIT, allergen-specific immunotherapy; APC, antigen presenting cells; cMono, classical monocytes; DC, dendritic cells; ILC, innate lymphoid cells; NK, natural killer cells; NKT, natural killer T cells; TCM, central memory T cells; CD8^{+} Tc1, type 1 cytotoxic cells; CD8^{+} Tc2, type 2 cytotoxic cells; Tc1-Tc17, hybrid type 1 and type 17 cytotoxic CD8^{+} T cells; Th1-Th17, hybrid type 1 and type 17 CD4^{+} T helper cells; TEM, effector memory T cells; TEMRA, terminally-differentiated effector memory T cells; Tn, naïve T cells; Treg, regulatory T cells. CyTOF, mass cytometry; PCA, Principal component analysis. For all the panels except for **G**, HC, healthy controls (n=10 independent individuals); PAP, pollen allergy patients (n=16 independent individuals); VAP, venom allergy patients (n=18 independent individuals). Source data are provided as a Source Data file.

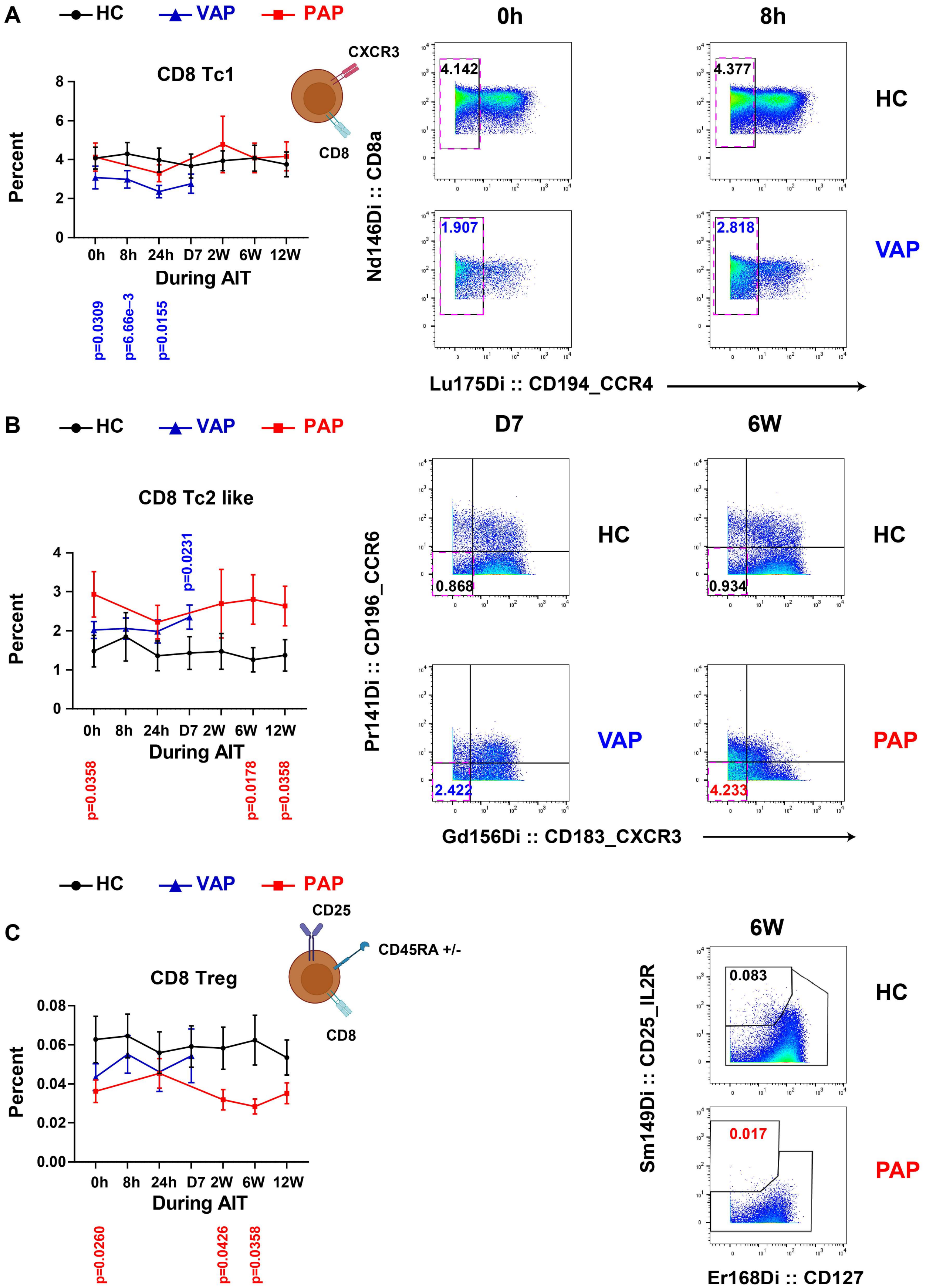
A
HC
VAP
PAP
CD8 Tc1
CXCR3
CD8
Percent
0h 8h 24h D7 2W 6W 12W
During AIT
p=0.0309
p=6.66e−3
p=0.0155
0h
8h
4.142
4.377
HC
1.907
2.818
VAP
Nd146Di :: CD8a
Lu175Di :: CD194_CCR4
B
CD8 Tc2 like
p=0.0231
p=0.0358
p=0.0178
p=0.0358
D7
6W
0.868
0.934
2.422
4.233
Pr141Di :: CD196_CCR6
Gd156Di :: CD183_CXCR3
C
CD8 Treg
CD25
CD45RA +/-
p=0.0260
p=0.0426
p=0.0358
0.083
0.017
Sm149Di :: CD25_IL2R
Er168Di :: CD127

Supplementary Fig. 3. $CD8^+$ subset dynamic responses in allergy patients during the initiation phase of AIT.

A-C, Dynamical patterns of the percentages of $CD8^+$ Tc1 (**A**), $CD8^+$ Tc2-like (**B**) or $CD8^+$ Tregs (**C**) among total living singlets. Right panel, Representative cytometry plots of the indicated markers from VAP and/or PAP or HC at the indicated time points. Dashed purple frame represents the target gate. For $CD8^+$ Tc1 ($CD8^+$ non-Treg then $CD45RA^-CD45RO^+CCR6^-CXCR3^+CCR4^-$): q=0.0416 (0h), q=0.0269 (8h), q=0.0314 (D1) between VAP and HC (**A**); for the non-Treg gate, please refer to Supplementary Fig.1. For $CD8^+$ Tc2 like ($CD8^+$ non-Treg then $CD45RA^-CD45RO^+CCR6^-CXCR3^-$): q=0.0932 (D7) between VAP and HC (**B**); q=0.0603 (0h), q=0.0603 (W6), q=0.0603 (W12) between PAP and HC (**B**); For $CD8^+$ Tregs ($CD8^+CD25^{high}CD127^{low}$): q=0.0717 (0h), q=0.0717 (W2), q=0.0717 (W6) between PAP and HC (**C**). Data represent mean± SEM. P-value was determined by non-paired two-tailed Mann-Whitney test (**A-C**). q values were generated using the two-stage step-up method (Benjamini, Krieger, and Yekutieli). The icon for the immune subset and its major markers used in our deep immunophenotyping analysis was provided for all the panels except for **B**. Unlabeled, not significant (p>0.05). AIT, allergen-specific immunotherapy; $CD8^+$ Tc1/2, type 1/2 cytotoxic cells; $CD8^+$ Tregs, $CD8^+$ regulatory T cells. For all the panels, HC, healthy controls (n=10 independent individuals); PAP, pollen allergy patients (n=16 independent individuals); VAP, venom allergy patients (n=18 independent individuals). Source data are provided as a Source Data file. Created in BioRender. Demczuk, A. (2024) BioRender.com/g57p989 (panel **A**, **C**).

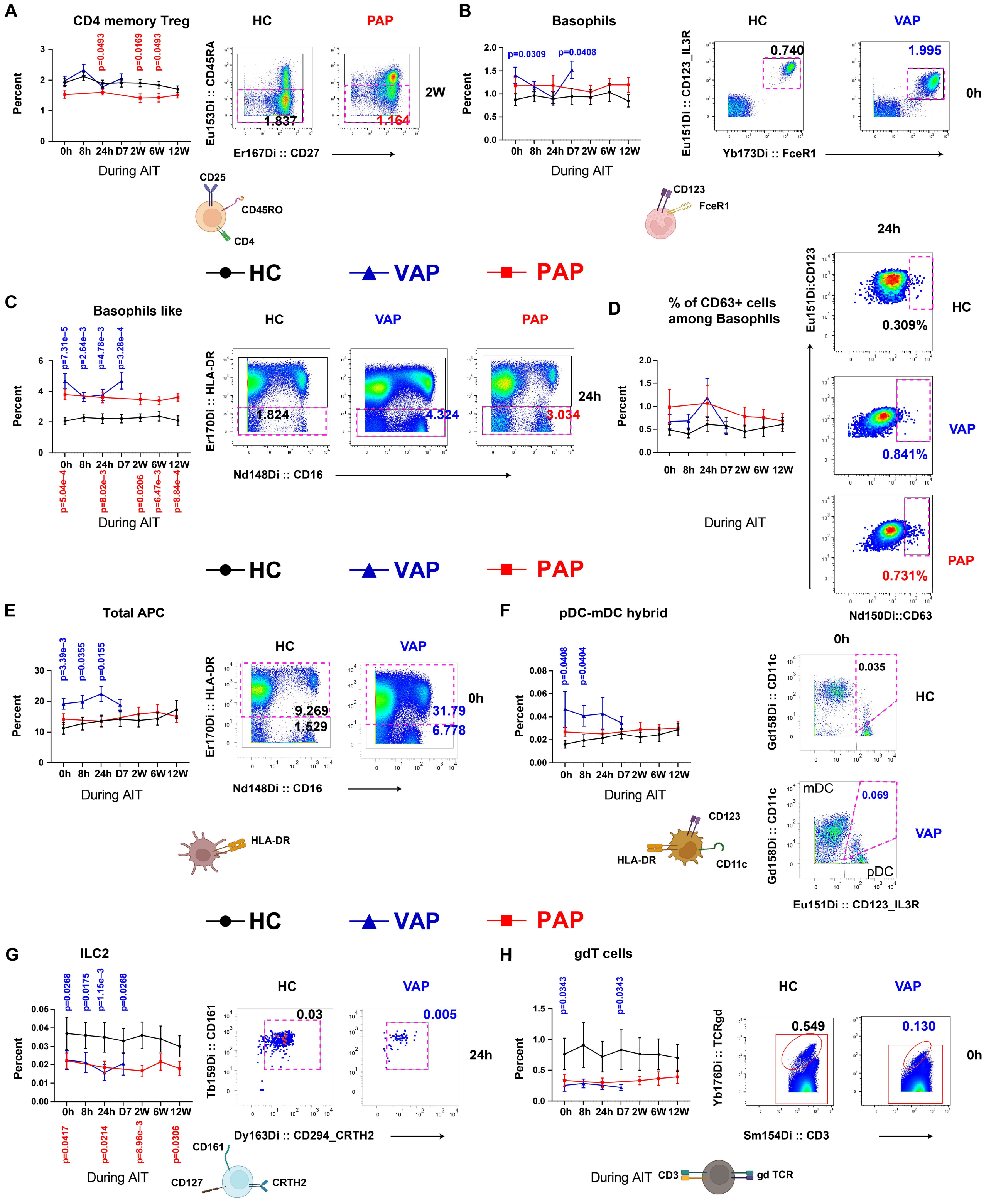

Supplementary Fig. 4. Early dynamic responses of other peripheral immune subsets in allergy patients following AIT launch.

A-C, **E-H**, Percentages of $CD4^+$ memory Tregs (**A**), Basophils (**B**), Basophils-like (**C**), total APC (**E**), pDC-mDC (**F**), ILC2 (**G**) or γδ T cells (**H**) among total living singlets of the participants from various groups (VAP, PAP and HC). **D**, Percentages of $CD63^+$ cells among basophils. Right panel,

Representative cytometry plots of the expression of indicated markers from VAP (and/or PAP) and HC at 24h. Dashed purple frame represents the target gate. CD4^{+} memory Tregs (CD3^{+}γδ T^{-}CD4^{+}CD25^{high}CD127^{low}CD45RA^{-}): q=0.0830 (24h), q=0.0830 (W2), q=0.0830 (W6) between PAP and HC (**A**); Basophils (CD3^{-}CD56^{-}CD19^{-}CD20^{-}HLA-DR^{-}CD123^{+}FceR1^{+}): q=0.0825 (0h) and q=0.0825 (D7) between VAP and HC (**B**); Basophils like (CD3^{-}CD56^{-}CD19^{-}CD20^{-}HLA-DR^{-}): q=2.95e-4 (0h), q=3.55e-3 (8h), q=4.83e-3 (24h), q=6.62e-4 (D7) between VAP and HC (**C**); q=1.34e-3 (0h), q=6.08e-3 (D1), q=0.0125 (W2), q=6.08E-3 (W6), q=1.34e-3 (W12) between PAP and HC (**C**); Total APC (CD3^{-}CD56^{-}CD19^{-}CD20^{-}HLA-DR^{+}): q=0.0137 (0h), q=0.0478 (8h), q=0.0314 (24h) between VAP and HC (**E**); hybrid pDC-mDC cells: (CD3^{-}CD56^{-}CD19^{-}CD20^{-}HLA-DR^{+}CD14^{-}CD16^{-}CD123^{+}CD11c^{+}) among total living singlets. q=0.0825 (0h), q=0.0825 (8h) between VAP and HC (**F**); ILC2 (CD4^{-}CD8^{-}CD14^{-}CD16^{-}CD123^{-}CD11c^{-}FceR1^{-}CD127^{+}CD161^{+}CRTH2^{+}): q=0.0203 (0h), q=0.0203 (8h), q=3.49e-3 (24h) q=0.0203 (D7) between VAP and HC (**G**); q=0.0527 (0h), q=0.0515 (24h), q=0.0452 (W2), q=0.0515 (W12) between PAP and HC (**G**); γδ T cells (CD3^{+}γδT^{+}): q=0.0559 (0h), q=0.0559 (8h), q=0.0559 (24h), q=0.0559 (D7) between VAP and HC (**H**). Data represent mean± SEM. P-value was determined by non-paired two-tailed Mann-Whitney test (**A-H**). q values were generated using two-stage step-up method (Benjamini, Krieger, and Yekutieli). Unlabelled, not significant (p>0.05). AIT, allergen-specific immunotherapy; APC, antigen presenting cells; ILC2, type 2 innate lymphoid cells; γδ T cells, gamma-delta T cells; pDC-mDC, hybrid plasmacytoid and myeloid DC; Treg, regulatory T cells. For panel **A-G**, HC, healthy controls (n=10 independent individuals); PAP, pollen allergy patients (n=16 independent individuals); VAP, venom allergy patients (n=18 independent individuals). For γδ T cells in panel **H**, due to staining issues the analyzed sample size at various time points is very different from that of other cell types (for the precise n number at different time points, please refer to Source Data and i3Dare). Source data are provided as a Source Data file. Created in BioRender. Demczuk, A. (2024) BioRender.com/x44j225 (panel **A**, **B**, **E-H**).

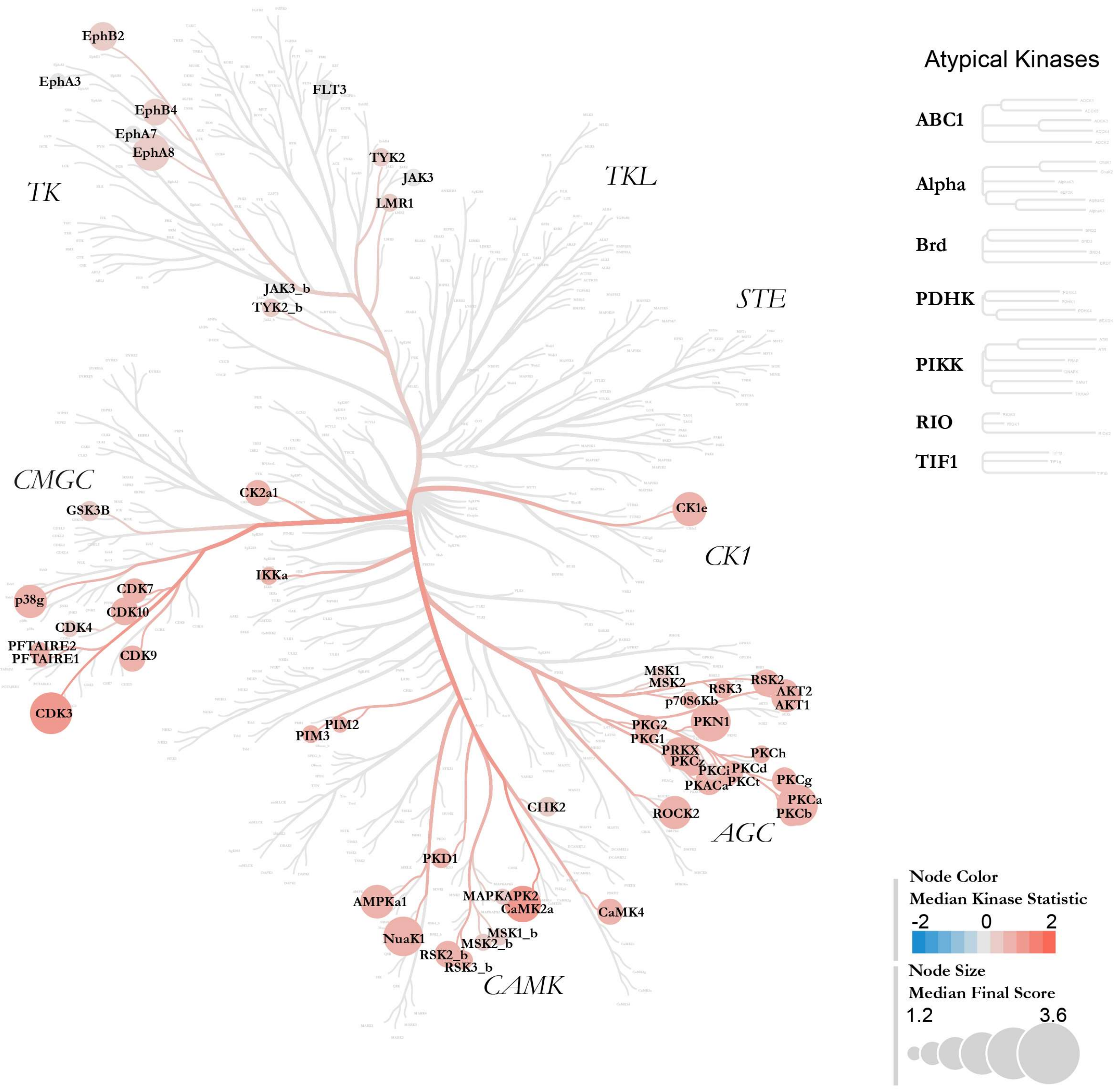

Supplementary Fig. 5. Human kinome analysis in VAP at 8h following AIT launch.

The PamGene kinome tree showed significantly changed kinases in PBMC depleted of $CD4^{+}$ T cells in VAP at 8h vs. baseline immediately following AIT launch. In total, ~ 350 unique human kinases were measured. Only the nodes showing a Median Final Score (representing confidence of the observation) >1.2 (the typical PamGene threshold) were marked with enlarged kinase label(s). The other kinases were only shown in small gray labels. The Median Kinase Statistic represents the direction of effect. Selected participants per group (n=9 independent participants; for the selection rationale, refer to Methods). For a zoomed-in view, please refer to Figure 4. For analysis details, refer to Methods. AIT, allergen-specific immunotherapy; VAP, venom allergy patients.

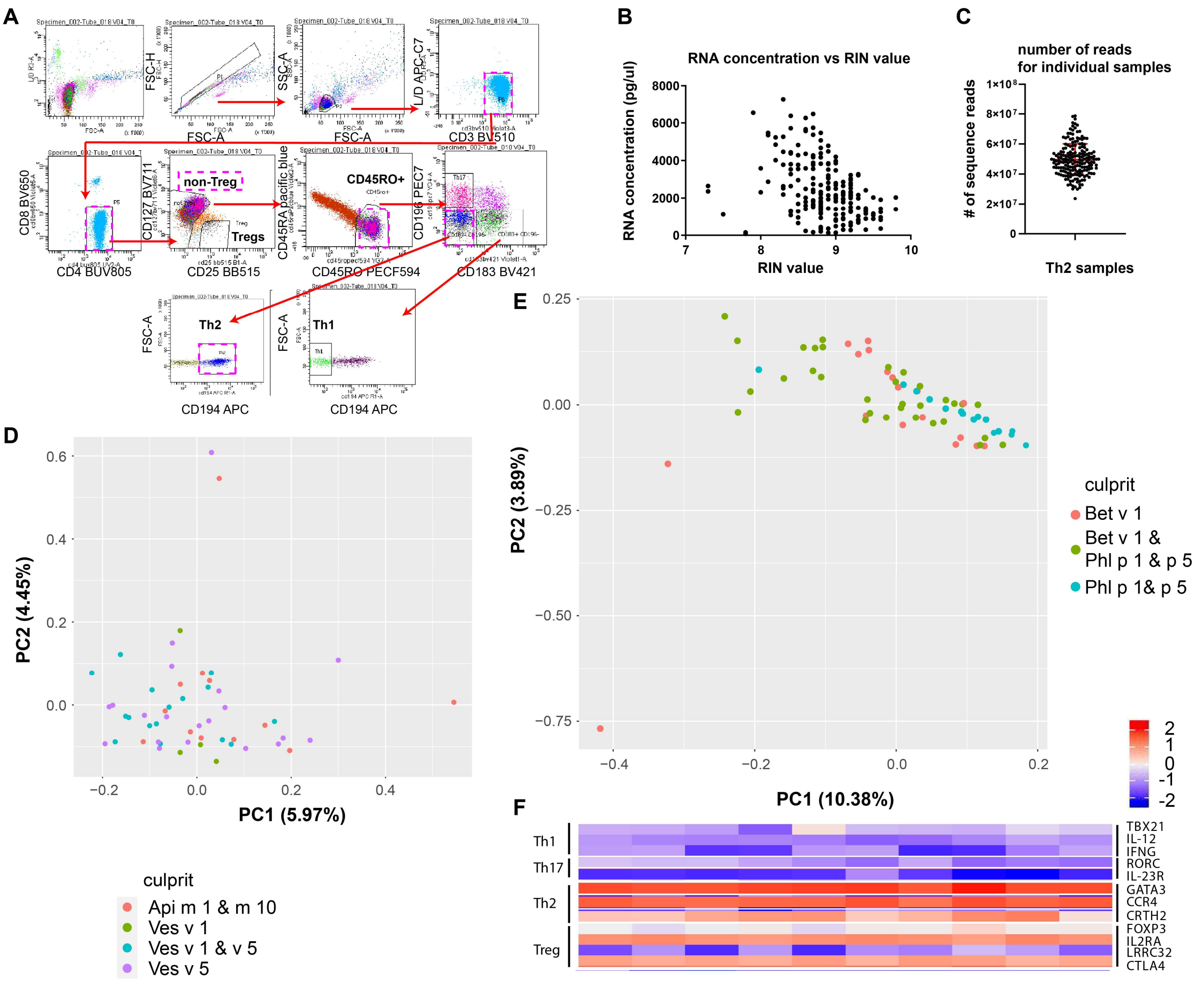

Supplementary Fig. 6. High-quality Th2-cell-type-specific RNA-seq analysis.

A, FACS-sorting strategy to define living CD4$^+$ Th2 singlets (CD3$^+$CD4$^+$CD8$^-$ non-Treg then CD45RO$^+$CD45RA$^-$CD183/CXCR3$^-$CD196/CCR6$^-$CD194/CCR4$^+$). For the non-Treg gate, please refer to Supplementary Fig.1. For detailed marker and sorting configuration information, please refer to Supplementary Table 4. Of note, our specific configuration using both V460/36 and V427/10 in our sorter allowing us to distinguish the fluorochromes pacific blue and BV421. Dashed purple rectangle was used to highlight a few gates or subsets. **B**, RNA integrity number (RIN) vs. RNA concentration of each individual RNA-seq sample (n=201 independent biological replicates). Each dot represents one individual sample. **C**, Distribution of the number of sequence reads (~50 million reads/sample) of individual Th2 RNA-seq samples (mean±SD) (n=187 independent biological replicates). **D, E**, PCA plot using the genome-scale RNA-seq datasets shows that there existed no culprit-allergen bias in the dataset of VAP (**D**) or PAP (**E**). **F**, Heatmap showing that known Th2-

specific (but not Th1- , Th17- or Treg-specific) marker genes were highly expressed in 10 randomly-selected independent biological samples. FACS, fluorescence-activated Cell Sorting; PCA, Principal component analysis; PC1/2, the first or second principal component. PAP, pollen allergy patients; VAP, venom allergy patients. Th1, $CD4^+$ type 1 helper T cells; Th2, $CD4^+$ type 2 helper T cells; Th17, $CD4^+$ type 17 helper T cells; Treg, $CD4^+$ regulatory T cells. Source data are provided as a Source Data file.

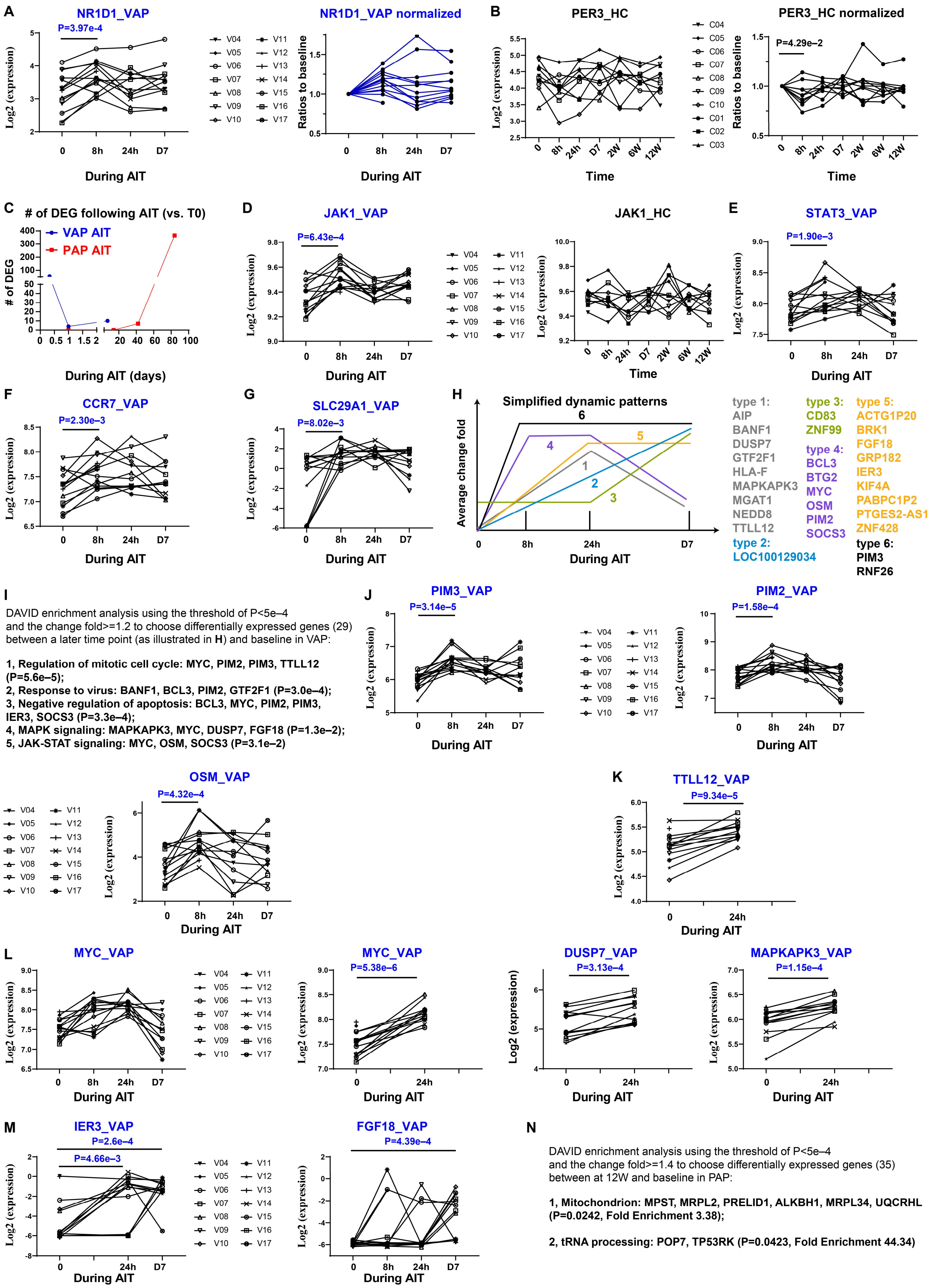
A
NR1D1_VAP
P=3.97e-4
Log2 (expression)
During AIT
NR1D1_VAP normalized
Ratios to baseline
B
PER3_HC
Time
PER3_HC normalized
P=4.29e−2
C
# of DEG following AIT (vs. T0)
VAP AIT
PAP AIT
# of DEG
During AIT (days)
D
JAK1_VAP
P=6.43e−4
JAK1_HC
E
STAT3_VAP
P=1.90e−3
F
CCR7_VAP
P=2.30e−3
G
SLC29A1_VAP
P=8.02e−3
H
Simplified dynamic patterns
Average change fold
type 1:
AIP
BANF1
DUSP7
GTF2F1
HLA-F
MAPKAPK3
MGAT1
NEDD8
TTLL12
type 2:
LOC100129034
type 3:
CD83
ZNF99
type 4:
BCL3
BTG2
MYC
OSM
PIM2
SOCS3
type 5:
ACTG1P20
BRK1
FGF18
GRP182
IER3
KIF4A
PABPC1P2
PTGES2-AS1
ZNF428
type 6:
PIM3
RNF26
I
DAVID enrichment analysis using the threshold of P<5e−4
and the change fold>=1.2 to choose differentially expressed genes (29)
between a later time point (as illustrated in H) and baseline in VAP:
1, Regulation of mitotic cell cycle: MYC, PIM2, PIM3, TTLL12 (P=5.6e−5);
2, Response to virus: BANF1, BCL3, PIM2, GTF2F1 (P=3.0e−4);
3, Negative regulation of apoptosis: BCL3, MYC, PIM2, PIM3, IER3, SOCS3 (P=3.3e−4);
4, MAPK signaling: MAPKAPK3, MYC, DUSP7, FGF18 (P=1.3e−2);
5, JAK-STAT signaling: MYC, OSM, SOCS3 (P=3.1e−2)
J
PIM3_VAP
P=3.14e−5
PIM2_VAP
P=1.58e−4
OSM_VAP
P=4.32e−4
K
TTLL12_VAP
P=9.34e−5
L
MYC_VAP
MYC_VAP
P=5.38e−6
DUSP7_VAP
P=3.13e−4
MAPKAPK3_VAP
P=1.15e−4
M
IER3_VAP
P=2.6e−4
P=4.66e−3
FGF18_VAP
P=4.39e−4
N
DAVID enrichment analysis using the threshold of P<5e−4
and the change fold>=1.4 to choose differentially expressed genes (35)
between at 12W and baseline in PAP:
1, Mitochondrion: MPST, MRPL2, PRELID1, ALKBH1, MRPL34, UQCRHL (P=0.0242, Fold Enrichment 3.38);
2, tRNA processing: POP7, TP53RK (P=0.0423, Fold Enrichment 44.34)

Supplementary Fig. 7. Extended Th2-cell-type-specific RNA-seq analysis.

A, mRNA expression of *NR1D1* in sorted Th2 cells. Right, Expression of *NR1D1* normalized to baseline. **B,** mRNA expression of *PER3*. Right, Expression of *PER3* normalized to baseline. **C**, Number of differentially-expressed genes (DEG) in Th2 cells at the given time point vs. baseline. **D**, mRNA expression of *JAK1* from VAP (left) or HC (right). **E, F, G**, mRNA expression of *STAT3* (**E**), *CCR7* (**F**) and *SLC29A1* (**G**). **H**, Six types of mRNA upregulation patterns in VAP following AIT. For the genes exhibiting a gradually-increasing pattern, the fold change between peak-time and baseline was at least 1.2 and showed a highly-significant P value (<=5e-4). For the genes exhibiting an increased plateau, the fold change between time points at the plateau and baseline was at least 1.2 and showed a highly-significant P value (<=5e-4) for at least one-time at the plateau. Of note, *S1PR1* was not listed here because it only peaked at 8h. **I,** **N**, DAVID enrichment analysis (https://david.ncifcrf.gov/tools.jsp, 2021) showing the top-ranked enriched pathways or processes among the genes with a significant change between a later time point (as defined in **H**) and baseline in VAP (**I**). The analysis was also performed for the upregulated genes (as defined in the panel) between W12 and baseline in PAP (**N**). **J**, mRNA expression of *PIM2*, *PIM3* and *OSM* from VAP. **K, L**, Uniform upregulation of *TTLL12* (**K**), *MYC*, *DUSP7* and *MAPKAPK3* (**L**) transcripts in VAP at 24h vs baseline. Of note, the expression of *MYC* was also displayed at all the time points during AIT (left). **M**, mRNA expression of *IER3* and *FGF18* within VAP. To avoid the message dilution, the values of *IER3* at 8h were not displayed. Samples from different time points of the same individual are connected with one line. P values in **A, B, D-G, J-M** were determined by paired two-tailed Student's *t* test. For all the panels, HC, healthy controls, n=10 independent individuals; PAP, pollen allergy patients, n=14 independent individuals; VAP, venom allergy patients, n=14 independent individuals. AIT, allergen-specific immunotherapy; Th2, $CD4^+$ type 2 helper T cells. Source data are provided as a Source Data file.

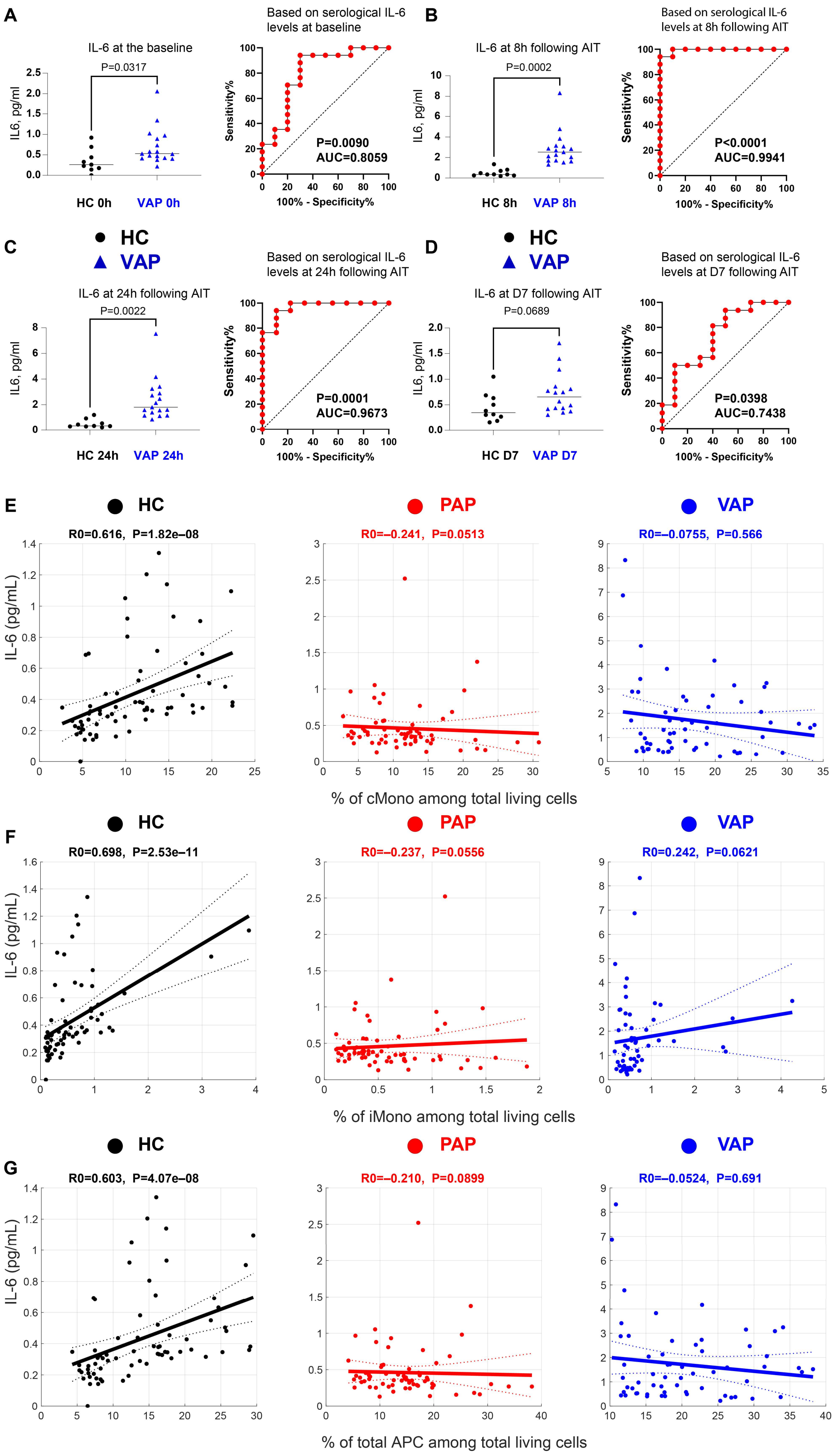
A
IL-6 at the baseline
P=0.0317
IL6, pg/ml
HC 0h
VAP 0h
Based on serological IL-6 levels at baseline
Sensitivity%
100% - Specificity%
P=0.0090
AUC=0.8059
B
IL-6 at 8h following AIT
P=0.0002
HC 8h
VAP 8h
Based on serological IL-6 levels at 8h following AIT
P<0.0001
AUC=0.9941
C
HC
VAP
IL-6 at 24h following AIT
P=0.0022
HC 24h
VAP 24h
Based on serological IL-6 levels at 24h following AIT
P=0.0001
AUC=0.9673
D
IL-6 at D7 following AIT
P=0.0689
HC D7
VAP D7
Based on serological IL-6 levels at D7 following AIT
P=0.0398
AUC=0.7438
E
HC
PAP
VAP
R0=0.616, P=1.82e–08
R0=–0.241, P=0.0513
R0=–0.0755, P=0.566
IL-6 (pg/mL)
% of cMono among total living cells
F
R0=0.698, P=2.53e–11
R0=–0.237, P=0.0556
R0=0.242, P=0.0621
% of iMono among total living cells
G
R0=0.603, P=4.07e–08
R0=–0.210, P=0.0899
R0=–0.0524, P=0.691
% of total APC among total living cells

Supplementary Fig. 8. Serological IL-6 is an early AIT-responsive biomarker in VAP and the balance between IL-6 and APC subsets are disturbed in both patient groups.

A-D, ROC analysis based on serological IL-6 levels at baseline (**A**, HC, n=10; VAP, n=17 independent individuals), 8h (**B**, HC, n=10; VAP, n=17 independent individuals), 24h (**C**, HC, n=9; VAP, n=17 independent individuals) or D7 (**D**, HC, n=10; VAP, n=16 independent individuals) following AIT launch in VAP vs IL-6 levels at the matched time points in HC without AIT administration. Left panel showing the group comparison at the indicated time point, where the median is labelled for each group. **E-G**, Spearman correlation between the percentages of cMono (CD14^{+}CD16^{-}, **E**, HC, n=69; VAP, n=60; PAP, n=66 independent biological samples), iMono (CD14^{dim}CD16^{dim}, **F,** HC, n=69; VAP, n=60; PAP, n=66 independent biological samples) or total APC (CD3^{-}CD56^{-}CD19^{-}CD20^{-}HLA-DR^{+}, **G,** HC, n=69; VAP, n=60; PAP, n=66 independent biological samples) and circulating IL-6 levels within HC, PAP and VAP. For the full gating strategy, please refer to Supplementary Fig.1 and the i3Dare website. The samples from all the time points were included in the analysis of the given group. P values were generated with a two-tailed exact permutation distribution test (**E-G**). P-value in the group comparison was generated using a two-tailed unpaired Student's *t* test while P-value from the ROC analysis tests the null hypothesis that the area under the curve really equals 0.50 with a two-tailed test (**A-D**). Each dot represents one independent biological sample at the given time point [the corresponding sample size n for each group is specified for each panel above]. AIT, allergen-specific immunotherapy; APC, antigen-presenting cells; cMono, classical monocytes; iMono, intermediate monocytes. HC, Healthy controls; PAP, pollen allergy patients; VAP, venom allergy patients. Source data are provided as a Source Data file.

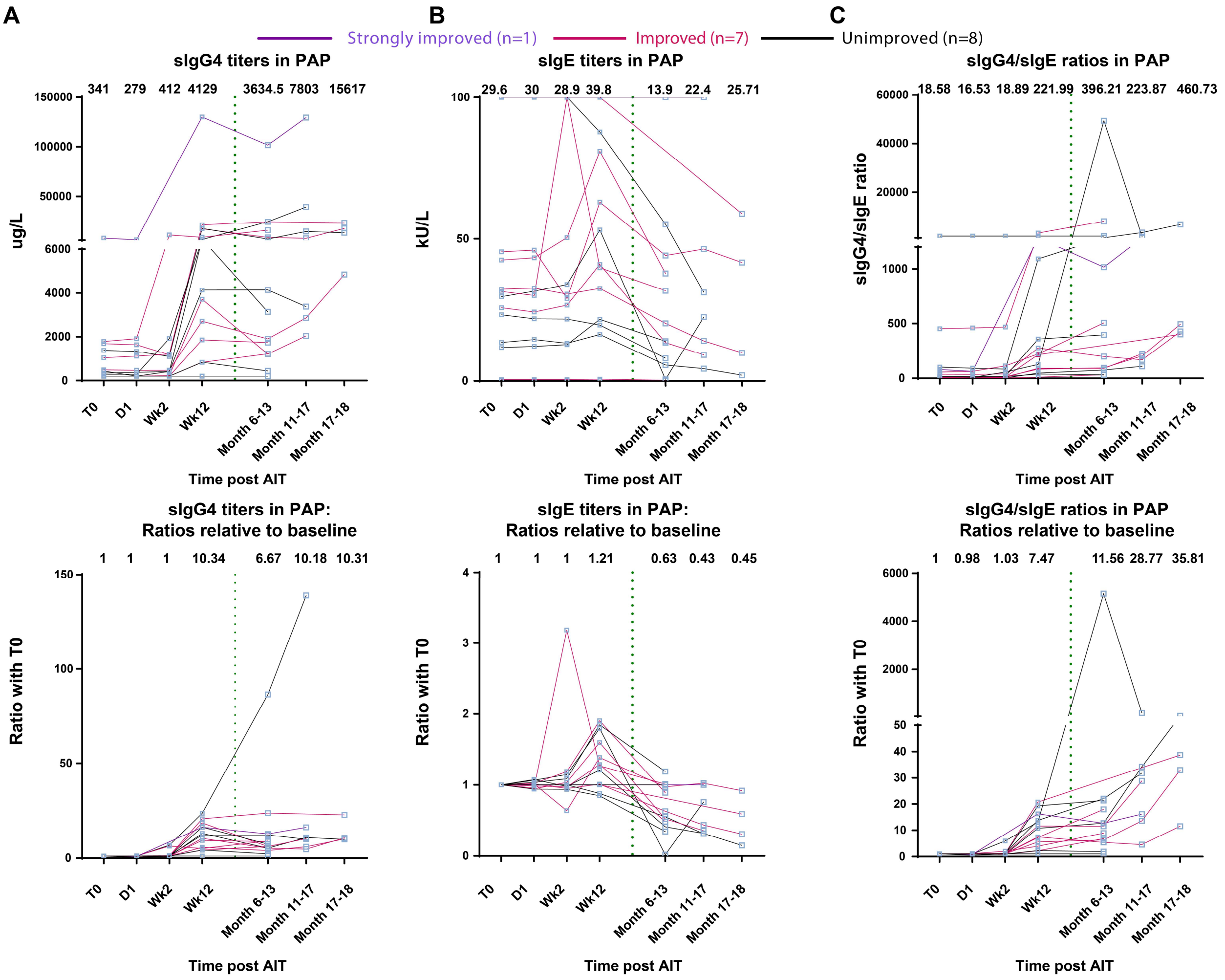

Supplementary Fig. 9. AIT induces varying sIgG4 responses in PAP independent of clinical outcomes.

A, B, Titers of sIgG4 (**A**) and sIgE (**B**) against birch pollen allergen rBet v 1 following AIT launch in pollen allergy patients (PAP). The dashed green line separates the long-term follow-up period from the early stage of AIT. Of note, the strongly improved patient in **B** had the highest sIgE titers (100) and showed a cut line in the upper panel. Lower panel in **A** and **B**, the values normalized to baseline. **C**, The ratios between sIgG4 and sIgE. Each line links different samples of one individual patient. Each rectangle represents one measurement of one individual at the given time point. Lower panel, the values normalized to baseline. The median response level for the samples at the given time point is provided. Strongly improved (n=1), improved (n=7) or unimproved (n=8) individual patients are indicated by different colors. Of note, one PAP did not show a sIgG4 response against rBet v 1 in the follow-up samples because the given patient was treated with grass pollen AIT (marked in Source Data). For another PAP also treated with grass pollen AIT, no long-term follow-up samples were available. AIT, allergen-specific immunotherapy. Source data are provided as a Source Data file.

Supplementary Tables:

Supplementary Table 1. Basic clinical and demographical information of the participants within the cohort.

	HC (median)	HC (IQR[£])	PAP (median)	PAP (IQR)	VAP (median)	VAP (IQR)	P-value between PAP and HC[€]	P-value between VAP and HC[€]
**Age (years)**	34.5	30-38	36.5	30.5-42.5	48.5	36.75-58.25	0.9036	0.009107
**BMI**	22.99	20.90-24.21	23.28	22.21-24.66	26.35	22.77-29.37	0.253391	0.067085
**Male%**	10		50		61.1			
**Smoking%**	80		6.25		44.4			
**Caucasian%**	100		81.25		100			
**Among 16 PAP, N (%)**								
**Initial diagnosis symptoms**								
**Seasonal Allergic rhinoconjunctivitis (SARC) alone**			6 (37.5)					
**SARC with Intermittent asthma (IAS) and Oral allergy syndrome (OAS)**			3 (18.75)					
**SARC with Intermittent asthma (IAS)**			3 (18.75)					
**SARC with Oral allergy syndrome (OAS)**			3 (18.75)					
**SARC with eosinophilic esophagitis (EOE)**			1(6.25)					
**Disease duration**								
**Disease duration 1-5 years**			9 (56.25)					
**Disease duration 5-10 years**			2 (12.5)					
**Disease duration >10 years**			5 (31.25)					
**Sensitizing allergens**								
**Sensitized to Bet v 1**			5 (31.25)					
**Sensitized to Phl p 1 and Phl p 5**			3 (18.75)					
**Sensitized to**			8 (50)					

**Bet v 1, Phl p 1 and Phl p 5**								
**AIT products**								
**AIT: Allergovit birch 100%**			4 (25)					
**AIT: Allergovit grass 100%**			3 (18.75)					
**AIT: Allergovit grass & birch**			9 (56.25)					
**AIT duration and outcomes**								
**Complete of full 3-5 year AIT**			15 (93.75)					
**Not improved (CSMS)[§]-like score, <10% score reduction)**			8 (50)					
**Improved (CSMS-like score, 10-25% score reduction)**			7 (43.75)					
**Strongly improved (CSMS-like score, >25% score reduction)**			1 (6.25)					
**Among 18 VAP, N (%)**								
**Initial diagnosis symptoms**								
**Anaphylaxis Grade 1 (Mueller Grading[γ])**					7 (38.89)			
**Anaphylaxis Grade 2**					7 (38.89)			
**Anaphylaxis Grade 3**					4 (22.22)			
**Sensitizing allergens**								
**Sensitized to Ves v 1**					1 (5.56)			
**Sensitized to Ves v 5**					8 (44.4)			
**Sensitized to both Ves v 1 and Ves v 5**					5 (27.78)			
**Sensitized to Api m 1**					1 (5.56)			
**Sensitized to Api m 1 and Api m 10**					3 (16.67)			
**AIT products**								
**AIT: Pharmalgen ALK wasp**					14 (77.8)			
**AIT: Pharmalgen ALK bee**					4 (22.2)			
**AIT duration and outcomes**								
**Complete of full 3-5 year AIT**					17 (94.44)			
**Field Sting(s) without systemic reaction**					9 out of 9 reported in 6 patients (100%)			
**Negative skin test at termination**					1 (not among the 6 field stung patients)			

Notes:

£, IQR: 25%-75% percentile (inclusive).

€, P-value was based on non-paired two-tailed Student's *t* test without the adjustment for multiple comparisons.

γ, Symptom severity degree defined by the 4-scale Mueller Grading; 4 indicates the most severe symptoms while 0 indicates no systemic reaction after a sting (for reference, please refer to Methods).

§, Combined Symptom Medication Score (CSMS) (for reference, please refer to Methods).

Supplementary Table 2. The list of CyTOF Abs used in this work.

Metal Isotope	target	Amount (µl)	Clone	Manufacturer	Catalogue	LOT (If more than one batch, we provide all)
**154Sm**	CD3	1	UCHT1	Fluidigm#	3154003B	0151806
**174Yb**	CD4	1	SK3	Fluidigm	3174004B	2351715
**146Nd**	CD8A	1	RPA-T8	Fluidigm	3146001B	1671716
**209Bi**	CD11B	1	ICRF44	Fluidigm	3209003B	0831723
**160Gd**	CD14	1	M5E2	Fluidigm	3160001B	3261702
**164Dy**	CD15 (SSEA-1)	1	W6D3	Fluidigm	3164001B	1421725
**148Nd**	CD16	1	3G8	Fluidigm	3148004B	2511709
**142Nd**	CD19	1	HIB19	Fluidigm	3142001B	0171815
**147Sm**	CD20	1	2H7	Fluidigm	3147001B	2491705
**166Er**	CD24	1	ML5	Fluidigm	3166007B	2651709
**149Sm**	CD25 (IL-2R)	1	2A3	Fluidigm	3149010B	1931712
**167Er**	CD27	1	L128	Fluidigm	3167006B	3501404
**172Yb**	CD38	1	HIT2	Fluidigm	3172007B	1931716
**171Yb**	CD44	0.1	IM7	Fluidigm	3171003B	1931725, 3421608
**89Y**	CD45	1	HI30	Fluidigm	3089003B	3421702
**165Ho**	CD45RO	1	UCHL1	Fluidigm	3165011B	1421721
**153Eu**	CD45RA	1	HI100	Fluidigm	3153001B	0641506, 641506
**155Gd**	CD56 (NCAM)	1	B159	Fluidigm	3155008B	2771704, 1471501
**150Nd**	CD63	1	H5C6	Fluidigm	3150021B	1801513
**152Sm**	CD66b	1	80H3	Fluidigm	3152011B	3491401
**144Nd**	CD69	1	FN50	Fluidigm	3144018B	3041705
**143Nd**	CD117 (cKit)	1	104D2	Fluidigm	3143001B	1711722

**151Eu**	CD123 (IL-3R)	1	6H6	Fluidigm	3151001B	2291711
**168Er**	CD127 (IL-7A)	1	A019D5	Fluidigm	3168017B	0541706
**145Nd**	CD138	1	DL-101	Fluidigm	3145003B	2651706
**159Tb**	CD161	1	HP-3G10	Fluidigm	3159004B	1361705
**156Gd**	CD183 (CXCR3)	1	G025H7	Fluidigm	3156004B	2771708
**175Lu**	CD194[&] (CCR4)	1	205410	Fluidigm	3175021A	2791705
**141Pr**	CD196 (CCR6)	1	11A9	Fluidigm	3141014A	0751705
**163Dy**	CD294 (CRTH2)	1	BM16	Fluidigm	3163003B	1671717, 691605
**170Er**	HLA-DR	1	L243	Fluidigm	3170013B	3571502
**161Dy**	CD152 (CTLA4)	1	14D3	Fluidigm	3161004B	2651711
**169Tm**	CD30*	1	81337	R&D	MAB229	Not available
**158Gd**	CD11C*	1	3.9	Biolegend	301639	Not available
**173Yb**	FceR1/ FCER1A*	1	AER-37 (CRA1)	Thermo Fisher Scientific	16-5899-82	Not available
**176Yb**	TCRγδ*	1	B1.1	Thermo Fisher Scientific	16-9959-81	Not available

Notes:

*, in-house conjugation using Maxpar X8 Antibody Labeling Kits.

&, this antibody was discontinued.

#, Fluidigm is now switched to Standard Bio.

Supplementary Table 3. The list of other CyTOF kits used in this work.

Kit Component	Manufacturer	Catalogue
**Maxpar Thulium Chloride 169Tm—50 mM**	Fluidigm	201169A
**Maxpar Gadolinium Chloride 158Gd—50 mM**	Fluidigm	201158A
**Maxpar Ytterbium Chloride 173Yb—50 mM**	Fluidigm	201173A
**Maxpar Ytterbium Chloride 176Yb—50 mM**	Fluidigm	201176A

Supplementary Table 4. The list of flow cytometry Abs and laser configuration used to sort Th2 cells of the participants.

Target	Alternative name	Fluorochrome	Clone	Used volume ul/10^6 cells	Catalogue, Manufacturer
**L/D**	Near IR for 633 or 635 Excitation			1	**L34976, Thermo Fisher Scientific**
**CD4**		BUV805	SK3	4	**564910, BD**
**CD183**	CXCR3	BV421	1C6/CXCR3	3	**562558, BD**
**CD45RA**		Pacific Blue	HI100	2	**304123, Biolegend**
**CD3**		BV510	HIT3a	3	**564713, BD**
**CD8**		BV650	RPA-T8	1	**301042, Biolegend**
**CD127**	IL-7R	BV711	A019D5	4	**351328, Biolegend**
**CD25**	IL-2RA	BB515	2A3	3	**564467, BD**
**CD45RO**		PE-CF594	UCHL1	2	**562299, BD**
**CD196**	CCR6	PE-Cy7	11A9	4	**560620, BD**
**CD194**	CCR4	APC	L291H4	2	**359408, Biolegend**

**Sorter configuration used for this project**		
**Excitation**	Denomination in DIVA	**Fluorochromes**
**UV Laser 355nm**	U785LP	**BUV805**
	U380/14	**BUV395**
**Violet Laser 405nm**	V775/50	**BV786**
	V730/45	**BV711**
	V661/11	**BV650**
	V610/20	**BV605/BV610**
	V525/50	**BV510**
	V460/36	**Pacific blue#**
	V427/10	**V450, BV421#**
**Blue Laser 488nm**	B710/50	**PerCP**
	B530/30	**FITC**
**Yellow Green Laser 561nm**	Y775/50	**PE-Cy7, PEVio770**
	Y670/14	**PE-Cy5**
	Y610/20	**PE-CF594**
	Y586/15	**PE**
**Red Laser 640nm**	R775/50	**APC-Cy7**
	R730/45	**Alexa Fluor 700**
	R670/14	**APC, Al647**

Note, #, the reason to provide the configuration setting is because we once used these two sorting markers with very close emission spectra for that specific project. For the gating strategy, please refer to Supplementary Fig. 6.
