# Supplementary material for: Multiomics approaches disclose very-early molecular and cellular switches during insect-venom allergen-specific immunotherapy: an observational study
Source: Nat Commun. 2024 Nov 26;15:10266. doi: 10.1038/s41467-024-54684-2 (PMC11599746; doi:10.1038/s41467-024-54684-2)
Supplement: Supplementary file 2 — Reporting Summary [file 41467_2024_54684_MOESM2_ESM.pdf]

nature portfolio

Corresponding author(s): Feng Q. Hefeng & Markus Ollert

Last updated by author(s): Oct 31, 2024

Reporting Summary

Nature Portfolio wishes to improve the reproducibility of the work that we publish. This form provides structure for consistency and transparency in reporting. For further information on Nature Portfolio policies, see our Editorial Policies and the Editorial Policy Checklist.

Statistics

For all statistical analyses, confirm that the following items are present in the figure legend, table legend, main text, or Methods section.

n/a	Confirmed	
☐	☒	The exact sample size (*n*) for each experimental group/condition, given as a discrete number and unit of measurement
☐	☒	A statement on whether measurements were taken from distinct samples or whether the same sample was measured repeatedly
☐	☒	The statistical test(s) used AND whether they are one- or two-sided<br>*Only common tests should be described solely by name; describe more complex techniques in the Methods section.*
☒	☐	A description of all covariates tested
☐	☒	A description of any assumptions or corrections, such as tests of normality and adjustment for multiple comparisons
☐	☒	A full description of the statistical parameters including central tendency (e.g. means) or other basic estimates (e.g. regression coefficient) AND variation (e.g. standard deviation) or associated estimates of uncertainty (e.g. confidence intervals)
☐	☒	For null hypothesis testing, the test statistic (e.g. *F*, *t*, *r*) with confidence intervals, effect sizes, degrees of freedom and *P* value noted<br>*Give P values as exact values whenever suitable.*
☒	☐	For Bayesian analysis, information on the choice of priors and Markov chain Monte Carlo settings
☒	☐	For hierarchical and complex designs, identification of the appropriate level for tests and full reporting of outcomes
☐	☒	Estimates of effect sizes (e.g. Cohen's *d*, Pearson's *r*), indicating how they were calculated

Our web collection on statistics for biologists contains articles on many of the points above.

Software and code

Policy information about availability of computer code

Data collection	Flow cytometry data were acquired using the BD FACSDiva Software (v8.0.1) and mass cytometry data were acquired using CyTOF Helios software v7.0. Cytokine data were recorded and analyzed on a MESO QuickPlex SQ 120 instrument (software version LSR_4_0_12). Kinome data were obtained with BioNavigator software (v6.3.67.0) on PamStation.
Data analysis	Flow cytometry data were analyzed using FlowJo V10. Generated .fcs files were normalized with the HELIOS acquisition software (v 7.0) by using EQ beads as a standard. Unless otherwise specified, the indicated frequency of the given subset was always relative to the total living singlets (for the full gating strategy, please refer to Supplementary Figure 1). Statistical analysis of supervised CyTOF analysis was done in Qluocore Omics Explore v 3.8 (1). The non-paired two-tailed Mann-Whitney test was applied to compare the immune subsets between different groups at the given matched time point while paired two-tailed Mann-Whitney test was used to compare the AIT response or natural immune fluctuations between a later time point and baseline from the same patients or controls. The volcano plot was visualized in Graphpad Prism v9.0. Of note, due to technical staining issues, the number of samples used for TCRγδ analysis (n=149) was lower than that for the other immune subsets (n=199) (for details regarding TCRγδ staining on which sample, participant and time point, please refer to our i3Dare website).<br><br>Although our CyTOF data was essentially analyzed by the supervised manual gating as described above, we also independently validated our discoveries of several selected subsets of interest (only Fig. 3c, 3i) by our unsupervised clustering and visualization methods. The unsupervised clustering analysis was performed using the pre-gated living singlets. Pre-gating of identifying live singlets was done in FlowJo v10. Lineage markers used for clustering have been arcsin transformed using a co-factor of 5. Unsupervised clustering has been performed using GigaSOM (https://github.com/LCSB-BioCore/GigaSOM.jl). GigaSOM is a FlowSOM-based clustering and dimension reduction method, adapted for huge-scale data sets using the high performance computing system in the Julia programming language (v 1.4). Of note, the analysis and visualization was performed without down-sampling. To visualize rare populations (CRTH2+ Th2 cells and Th1-Th17 hybrid cells in Fig. 3c, 3i), we conducted

a sub-clustering on the extracted CD4+ T cells that were first manually exported from FCS files using Flowjo v10. The sub-clustering analysis utilized a 20x20 SOM-grid, 100 epochs and exponential radius decay using only cell type specific set of markers [CD196 (CCR6), CD25 (IL 2R), CD127 (IL 7Ra), CD45RA, CD45RO, CD183 (CXCR3), CD161, CD294 (CRTH2), CD194 (CCR4)]. Following clustering, cell type annotation was performed using the CyCadas tool (https://github.com/DII-LIH-Luxembourg/cycadas) with default settings. The plot was visualized using EmbedSOM v2 (https://github.com/exaexa/EmbedSOM). The recovered subsets were highlighted in a 2-dimensional embedding (Fig. 3c, 3i).

For the kinome analysis, from each PamGene chip, a series of images describing the phosphorylation of peptides (196 for PTK and 144 for STK) corresponding to ~350 native kinases in the applied lysate was obtained. Using the instrument manufacturer's , data was curated and normalized by chip using COMBAT before running an upstream kinase analysis. This analysis provides the Median Kinase Statistic describes the direction of effect (activation or inhibition): the change in kinase activity in the test condition (i.e., 8h following AIT) compared to a control condition (i.e., baseline) and is formed from the median of the peptide statistics of the set of peptides that a kinase has been associated to within a defined database. The analysis also generates the Median Final Score, the sum of the significance score and the specificity score each taking a Mixed Model statistical analysis based on a one-tailed permutation test without multiple comparison correction by randomly altering sample assignment and the peptide-kinase relationship assignment, respectively. R v4.1.1 was used to compile the results and to display them using an adaptation of the CORAL tool (https://github.com/dphansti/CORAL).

NULISAseq assay sequencing data were processed using the NULISAseq algorithm (Alamar Biosciences). The sample- and target-specific barcodes were quantified, and up to two mismatching bases or one indel and one mismatch were allowed. Intraplate normalization was performed by dividing the target counts for each sample well by that well's internal control counts. Interplate normalization was then performed using interplate control (IPC) normalization, wherein counts were divided by target-specific medians of the three IPC wells on that plate. Data were then rescaled. Following addition of +1, the data were log2 transformed to obtain NULISA Protein Quantification (NPQ) units for downstream statistical analysis.

Based on the corr function in MATLAB 2020a, the correlation coefficient matrixes within or between different datasets were calculated (e.g., within different immune subsets, between immune subsets and cytokines). Since the datasets often do not follow a normal distribution, the Spearman correlation coefficient was employed for the correlation analysis described in this section. The continuous range of correlation coefficients is (-1, +1). The P-values following the two-tailed test were also directly generated by the corr function. When the P-value is less than 0.05, we considered that the corresponding correlation coefficient was significantly different from zero.

The principal component analysis (PCA) was performed on the default setting of the pca function in MATLAB 2020a. Considering different types of datasets, different groups of participants and different sampling time points, we divided the analysis into two categories. One type was to analyze the sample distribution between different participant groups using different combinations of datasets at a fixed sampling time point; the second type was to analyze time evolution responses within a fixed participant group. Before the PCA analysis, all the data were centered by the zscore function so that each variable had a mean value of 0 and a standard deviation of 1. In order to compare the changes of the overall immune features of each patient following immunotherapy (or each healthy control following the sampling period), we calculated the average distance among pairs of the adjacent two time points of each participant in the two-dimensional PCA plots. The larger the distance is, the more the overall peripheral immune system of the given subject has changed over the sampling period either caused by immunotherapy and/or natural fluctuation. Of note, each plot in the time-slice PCA figure (Figure 2B and Supplementary Figure 2D) is independent and different PCA plots were draw together only for better visualization.

Quality control of the raw RNA sequencing data was conducted using the FastQC software (v0.11.7-Java-1.8.0_162) (https://www.bioinformatics.babraham.ac.uk/projects/fastqc/). Reads were mapped to the reference human genome 38 (http://hgdownload.soe.ucsc.edu/goldenPath/hg38/bigZips/) and summarized to genes using the R Bioconductor Rsubread package (v1.32.4). One sample was excluded for further analysis because CD127 Ab was forgotten to add before sorting. Clinical and demographic factors that are significantly different between groups can have a confounding effect on subsequent analyses if no correction for these factors is applied. The following statistical tests were conducted to identify potential confounding factors. For categorical variables, a Fisher's exact test was performed, whereas for normally distributed numerical variables having equal variances, one-way ANOVA was applied. In case of a normally distributed variable with unequal variances between the groups, Welch one-way test was used. For non-normally distributed values, the Kruskal-Walls rank sum test was employed. Homogeneity of variances was determined using Levene's test, while normality was tested using quantile-quantile (Q-Q) plots. Genes that consistently had zero or very low counts were first removed using the Bioconductor edgeR package (v3.24.3). Trimmed Mean of M-values (TMM) normalization was performed on the filtered data with the edgeR package. The normalized data were voom-transformed to enable differential expression analysis with the Bioconductor limma package (v3.38.3). Principal component analysis (PCA) was performed on the voom-transformed data, and a score plot was presented where each batch was represented by a different colored symbol. In case of no batch effect, the batches do not cluster in the PCA plot. Furthermore, a principal coordinate analysis (PCoA) was conducted with the R Stats package and the score plot was inspected to check for potential outliers. Differentially-expressed genes (DEGs) between allergic patients and controls, and between AIT-treated samples and baseline were determined by applying an empirical Bayes moderated t-test on the voom-transformed data, implemented in the R Bioconductor limma package (v3.38.3). Nominal p-values were corrected for multiple hypothesis testing by computing false discovery rates (FDR) according to the Benjamini-Hochberg procedure and using an FDR significance threshold of 0.05. Entrez gene IDs were converted to gene symbols with the R Bioconductor org.Hs.eg.db package (v3.7.0). DEGs were visualized in heatmaps and volcano plots using the R Bioconductor ComplexHeatmap (v1.20.0) and R ggplot2 (v3.2.0) packages, respectively. Enriched differentially-regulated pathways and GO (Gene Ontology) biological processes were determined by over-representation analysis in ConsensusPathDB (release 34, http://cpdb.molgen.mpg.de/) using all measured genes as background list. Pathways were selected from Reactome, KEGG, Wikipathways, Biocarta and PID. Pathways and GO biological processes with Benjamini-Hochberg corrected FDR ≤ 0.05 were considered significant. All the scripts used for the RNA-seq data analysis are deposited in Gitlab and accessible under accession code https://zenodo.org/records/13926742 (DOI: 10.5281/zenodo.13926742).

For manuscripts utilizing custom algorithms or software that are central to the research but not yet described in published literature, software must be made available to editors and reviewers. We strongly encourage code deposition in a community repository (e.g. GitHub). See the Nature Portfolio guidelines for submitting code & software for further information.

Data

Policy information about availability of data

All manuscripts must include a data availability statement. This statement should provide the following information, where applicable:

- Accession codes, unique identifiers, or web links for publicly available datasets
- A description of any restrictions on data availability
- For clinical datasets or third party data, please ensure that the statement adheres to our policy

All the raw and processed genome-scale Th2-specific RNA-seq data have been deposited in the GEO database with the accession number GSE240503 (https://www.ncbi.nlm.nih.gov/geo/query/acc.cgi?acc=GSE240503). All the raw fcs data of CyTOF analysis are deposited and available under restricted access for academic non-commercial research via (https://doi.org/10.5281/zenodo.14001917). The contacted corresponding author(s) will respond to the request within 10 working days and the shared link shall be expired within one month. For commercial access of our CyTOF data, parties will be directed to an appropriate contact. Our processed large-scale interactive interlinked immunological Data resource (i3Dare) of CyTOF data allows investigators to more effectively explore or reuse our immunological datasets of each immune subset from each participant at each time point (please refer to https://public.tableau.com/app/profile/lihpublicdata/viz/i3Dare_SYSTACT_Database/SYSTACTHome; ensure to activate the full-screen mode in the lower right corner for better navigation experience). Source data are provided with this paper. The disaggregated sex information is also available in Source Data.

Research involving human participants, their data, or biological material

Policy information about studies with human participants or human data. See also policy information about sex, gender (identity/presentation), and sexual orientation and race, ethnicity and racism.

Reporting on sex and gender	In the study design, we did not consider the sex ratio as the recruitment criteria for this real-world observational study. While our two patients groups (VAP and PAP) included male and female participants at almost 1:1 ratio (PAP: 8 males, 8 females; VAP: 11 males, 7 females), the HC group was predominantly female (9 of 10). Therefore, for our cohort, there is no suitable way for us to split the participants into male or female subgroups to perform sex-specific analysis (as HC were essentially females). Anyway, our cohort sample size also does not allow us to further split it into subgroups for a meaningful analysis. In Supplementary Table 1, we have provided the detailed sex distribution information for each participant group. Apparent gender was evaluated together with other biometric parameters as part of the routine physical examination by the clinical team before or during the recruitment process. The disaggregated sex information of each relevant panel of each relevant Figure is also available in Source Data.
Reporting on race, ethnicity, or other socially relevant groupings	The % of Caucasians in the participant groups: 100% (HC), 81.25% (PAP), and 100% (VAP).
Population characteristics	The distribution (mean and 25-75 percentile) of age and BMI as well as males%, smoking% and Causcasian% are provided in Supplementary Table 1. While the age of the PAP was comparable to that of HC (median, around 37 and 35 years, respectively, Supplementary Table 1), VAP were older (median, around 49 years). While both patient groups included male and female participants at almost 1:1 ratio, the HC group was predominantly female (9 of 10). Five of 16 PAP were sensitized to major birch pollen allergen (Bet v 1), three of 16 exhibited elevated sIgE and clinical reactivity to timothy grass pollen allergens (Phl p 1 and Phl p 5) and eight were allergic to the pollen of both species. Most VAP (14 of 18) presented with a history of systemic reactions to wasp (yellow jacket; Vespula spp.) stings, being sensitized to Ves v 1 (one of 18), Ves v 5 (eight of 18) or both allergens (five of 18). Three of four bee VAP showed elevated sIgE levels to both Api m 1 and Api m 5, another VAP being mono-sensitized to Api m 1. Two VAP are hobby beekeepers.
Recruitment	The study participants with moderate-to-severe allergic rhinitis (pollen allergy patients, PAP, n=16) and insect sting reactions (venom allergy patients, VAP, n=18) eligible for AIT in accordance with the current international guidelines were recruited at a tertiary center (Service National d'Immuno-Allergology of Centre Hospitalier de Luxembourg) between August 2016 and February 2018. The healthy controls (HC, n=10) were recruited at the Clinical and Epidemiological Investigation Center of the Luxembourg Institute of Health (CIEC-LIH) between Jan and Apr 2018. The study was designed as a real-world, prospective, exploratory, data-driven trial for identification of novel potential biomarkers and molecular switches in the early time window of AIT in the outpatient clinical setting. The inclusion criteria were the confirmed clinical diagnosis of allergy (skin prick test positivity and elevated sIgE titers to plant pollen or insect venom allergens, respectively), clinical indication for launch of AIT for the patients and absence of known allergies for healthy volunteers. The exclusion criteria were age less than 18 years, pregnancy and lactation; overt asthma or chronic obstructive pulmonary disease, concomitant autoimmune disorders, chronic diseases in exacerbation or not adequately controlled, history of hematological malignancies or solid tumors, treatment with systemic steroids, immunomodulatory agents or biologics, acute and exacerbation of chronic infections, traumas and surgeries in six months prior to the study enrollment. No restrictions were introduced regarding the number of sensitizing allergens, duration of the allergic disease, the time elapsed since the last field sting and treatment with mono- versus polyvalent allergen immunotherapy products. In VAP, an ultra-rush AIT protocol was administered to reach the maintenance dose within 8 hours with 14 subcutaneous injections (Figure 1A). As no comparable ultra-rush up-dosing AIT products were licensed for pollen allergy, neither at the start of the project nor subsequently, the exact sampling periods differed between VAP and PAP. For PAP, a conventional up-dosing AIT was applied to reach the maintenance dose within 6 weeks with a weekly subcutaneous injection scheme. Thus, our sampling schemes were critically chosen to cover comparable phases during the build-up of AIT until the targeted maintenance dose of allergens was achieved for both types of AIT. In this real-world study, no self-selection bias was introduced because no sex ratio, race and age of adults were considered during the recruitment stage.
Ethics oversight	This study complied with all ethical regulations for conducting studies with human participants and was approved by the Luxembourg National Research Ethics Committee (CNER). The study was approved by CNER (SYS-T-Act: No. 201509/11) and pre-registered with the study protocol at ClinicalTrials.gov on October 11th, 2016 (NCT02931955; https://clinicaltrials.gov/

study/NCT02931955). The experiments conformed to the principles set out in the World Medical Association (WMA) Declaration of Helsinki and the Department of Health and Human Services Belmont Report. Informed consent was obtained from each participant prior to sampling and personal data collecting, which were in full compliance with the Luxembourg National Commission for Data Protection (CNPD, before the implementation of GDPR in Europe). We obtained notification on 11th Sep, 2015 and modification approval on 17th Jul, 2017 from CNPD.

No financial reward was given for VAP or PAP. Each of the ten HC received a compensation of 175 euro for their time to participate this study (to be sampled seven times within a three-month period) and of 25 euro for their travel costs.

Note that full information on the approval of the study protocol must also be provided in the manuscript.

Field-specific reporting

Please select the one below that is the best fit for your research. If you are not sure, read the appropriate sections before making your selection.

[x] Life sciences [ ] Behavioural & social sciences [ ] Ecological, evolutionary & environmental sciences

For a reference copy of the document with all sections, see nature.com/documents/nr-reporting-summary-flat.pdf

Life sciences study design

All studies must disclose on these points even when the disclosure is negative.

Sample size

In our study, no sample size power calculation was performed in advance.

At the outpatient facility of the national unit of Immunology-Allergology (Service National d'Immuno-Allergology of Centre Hospitalier de Luxembourg) which treats a great variety of allergic and immune diseases, approximately 30 to 40 new patients for anaphylactic reactions to Hymenopter stings and around 1000 new patients for seasonal pollinosis symptoms are seen every year. After general physical examination, control of biometric data, skin and specific blood IgE testing, different treatment options are discussed with the patients. Finally, approximately half of the patients with anaphylactic reactions to wasp or bee stings will have immunotherapy following an ultra-rush protocol, and about half the patients with pollinosis symptoms will have immunotherapy to grass- or tree-pollen. Of these the great majority of the pollen patients will have sublingual immunotherapy and only around ten percent will follow a sub-cutaneous up-dosing pre-seasonal protocol. In this way, a sample size of 15 per patient group was determined empirically as an achievable recruitment target at the given single clinical center over a one-year period. Eventually, the study includes 18 (VAP) venom allergic patients following an ultra-rush protocol, 16 (PAP) pollen-allergic patients following a conventional up-dosing subcutaneous AIT protocol and 10 healthy controls (HC). The study participants with moderate-to-severe allergic rhinitis (pollen allergy patients, PAP, n=16) and insect sting reactions (venom allergy patients, VAP, n=18) eligible for AIT in accordance with the current international guidelines were recruited at Service National d'Immuno-Allergology of Centre Hospitalier de Luxembourg between August 2016 and February 2018. According to the original plan, we only need to recruit 15 VAP. Due to the technical issue faced during cell sorting in the first three VAP, we recruited three more VAP and eventually ended up with 18 VAP. For the first three VAP, whenever possible, different layers of analyses except for RNA-seq in sorted Th2 cells were still performed. The healthy controls (HC, n=10) were recruited at the Clinical and Epidemiological Investigation Center of the Luxembourg Institute of Health (CIEC-LIH) between Jan and Apr 2018. The sample size of 10 in HC was determined empirically in consideration of the feasibility to recruit enough healthy volunteers who are willing to be sampled seven times within a 3-month period.

Although no prior power calculation was made, our hypothesis-free deep immunophenotyping study of >70 different immune subsets still provided sufficient power to detect the changes of some immune subsets as several cell types indeed showed significant observations.

Data exclusions

Essentially, no data exclusion was done, except for the specified technical failures during the staining.

Replication

For the venom allergic patients (VAP), n=18; for the Pollen allergic patients (PAP), n=16; for the healthy controls (HC), n=10. Each participant represents one independent biological replicate. In the meantime, each participant has been sampled longitudinally overtime (up to seven times within a three-month period for HC). In total, we analyzed up to 200 different peripheral blood biological samples from our cohort. We only reported those showing statistical significant results in the Main or Supplementary Figures by comparing all the measured samples or participants between different groups, either at the same time point or at a later time point versus baseline within one group.

We want to highlight that for some of the results, for example, serological IL-6 increased temporarily at 8h vs baseline among almost all the VAP. Transcription expression of SOCS3 was temporally increased uniformly at 8h vs baseline among all the individual VAP.

The cytokine data (essentially IL-6 results) have been successfully repeated by two different investigators either in the beginning of the project or in the end of the project period, independently. The key finding of increased non-inflammatory IL-6 levels in the VAP patients upon AIT launch was further validated using another type of assay (NULISAseq assay, Alamar).

The CyTOF experiments were performed in an ISO9001:2015 certified Luxembourg National Cytometry Platform. Technically, we have also obtained the antibody specificity certificate for each of the used CyTOF abs from the provider. We have verified the reproducibility of our major immunological data in either 18 independent VAP patients or 16 independent PAP patients at different time points vs. 10 independent healthy controls (HC) at different time points. Furthermore, the confidence of our data was validated across different immune subsets that are supposed to play a role together.

For the large-scale CyTOF, RNA-seq data, NULISAseq and Kinome analysis, there were no technique replicates, but all the patients or healthy controls represent independent biological replicates.

Randomization

Randomization is impossible because we had to implement all the stringent inclusion and exclusion criteria to recruit the two groups of

allergic patients for AIT treatment or HC participants. Furthermore, we could not interfere and decide when the selected participants would visit the clinical team (for patients) to receive the AIT treatment or research nurses (for healthy controls).

Blinding

The experimenters performing sorting were not blinded to the group allocation during data collection because an pseudoID without disclosing any personal identity information (e.g., V01T02, P01T02 or HC01T02; '01' is an exemplary ID following the letter "V" or "P" or "C" and '02' is an exemplary time point following the label "T") had to be first assigned to each participant for the given time point by the research clinician. However, we do not think this will affect our results as the essential quantification equipment (Cytometry) acquired immune cells unbiasedly (i.e., the investigators cannot influence the objective machine measurements/readouts on each of a large number of analyzed immune subsets for the given participant at the given time point).

For CyTOF analysis, the experimental operators were blinded to the group and time point information (they only knew the sample kit ID, e.g., 12345678, which is completely different from the sample pseudoID). Furthermore, the CyTOF staining/analyzing investigators were even different from the mass spectrometry Helios operators who acquired all the cells and initially normalized the data.

For cytokine multiplexing assay experiments, one also cannot influence the readouts of the corresponding quantification machine/platform (MSD platform used). For RNA-seq analysis, the people who performed sorting were different from those who prepared RNA extraction, different from those who performed sequencing, and also completely different from those who performed computational analysis (even from another cooperation partner). Moreover, none of the operators can influence the objective readouts of their responsible steps. The operators performing NULISA assays were completely blinded to the sample time points and group information. The clinical laboratory technicians performing sIgG4 and sIgE were also completely unaware of any sample group information. Furthermore, the operators (for both NULISA and sIgG4 and sIgE measurements) cannot influence the machine readouts in a sample-specific manner.

For the Kinome analysis, the operator was also not blind to the sample ID as a group balanced design has to be adopted for each chip. A separate aliquot of cell lysate was assessed on the PTK chip (86402, PamGene) and the STK chip (87102, PamGene) using the manufacturer's protocol. From each chip, a series of images describing the phosphorylation of peptides (196 for PTK and 144 for STK) corresponding to ~350 native kinases in the applied lysate was obtained. The analyzer cannot control and affect the readout as well.

In short, applying a blinding analysis or not has no impact on our various machine-based objective measurements, analyses and conclusions in this work.

Reporting for specific materials, systems and methods

We require information from authors about some types of materials, experimental systems and methods used in many studies. Here, indicate whether each material, system or method listed is relevant to your study. If you are not sure if a list item applies to your research, read the appropriate section before selecting a response.

Materials & experimental systems

n/a	Involved in the study
☐	☒ Antibodies
☒	☐ Eukaryotic cell lines
☒	☐ Palaeontology and archaeology
☒	☐ Animals and other organisms
☐	☒ Clinical data
☒	☐ Dual use research of concern
☒	☐ Plants

Methods

n/a	Involved in the study
☒	☐ ChIP-seq
☐	☒ Flow cytometry
☒	☐ MRI-based neuroimaging

Antibodies

Antibodies used

All the used mass and flow cytometry antibodies (Abs) including the provider, cat number, clone, dilution factor or Ab amount, specific metal isotype for mass cytometry or specific fluochromes for flow cytometry were already included in Supplementary Table 2, 3 and 4. We listed them here again.

List 1 (Supplemental Table 2). The list of CyTOF Abs used in this work:
Metal Isotope, Antibody, Ab Amount (µl), Clone, Manufacturer, Catalogue#, LOT# (whenever available; if more than one LOT was used, they are split by '&')
154Sm CD3, 1, UCHT1, Fluidigm, 3154003B, 0151806
174Yb CD4, 1, SK3, Fluidigm, 3174004B, 2351715
146Nd CD8a, 1, RPA-T8, Fluidigm, 3146001B,1671716
209Bi CD11b, 1, ICRF44, Fluidigm, 3209003B, 0831723
160Gd CD14, 1, M5E2, Fluidigm, 3160001B, 3261702
164Dy CD15 (SSEA-1), 1, W6D3, Fluidigm, 3164001B, 1421725
148Nd CD16, 1, 3G8, Fluidigm, 3148004B, 2511709
142Nd CD19, 1, HIB19, Fluidigm, 3142001B, 0171815
147Sm CD20, 1, 2H7, Fluidigm, 3147001B, 2491705
166Er CD24, 1, ML5, Fluidigm, 3166007B, 2651709
149Sm CD25 (IL-2R), 1, 2A3, Fluidigm, 3149010B, 1931712

167Er CD27, 1, L128, Fluidigm, 3167006B, 3501404
172Yb CD38, 1, HIT2, Fluidigm, 3172007B, 1931716
171Yb CD44, 0.1, IM7, Fluidigm, 3171003B, 1931725 & 3421608
89Y CD45, 1, HI30, Fluidigm, 3089003B, 3421702
165Ho CD45RO, 1, UCHL1, Fluidigm, 3165011B, 1421721
153Eu CD45RA, 1, HI100, Fluidigm, 3153001B, 0641506 & 641506
155Gd CD56 (NCAM), 1, B159, Fluidigm, 3155008B, 2771704 & 1471501
150Nd CD63, 1, H5C6, Fluidigm, 3150021B, 1801513
152Sm CD66b, 1, 80H3, Fluidigm, 3152011B, 3491401
144Nd CD69, 1, FN50, Fluidigm, 3144018B, 3041705
143Nd CD117 (cKit), 1, 104D2, Fluidigm, 3143001B, 1711722
151Eu CD123 (IL-3R), 1, 6H6, Fluidigm, 3151001B, 2291711
168Er CD127 (IL-7a), 1, A019D5, Fluidigm, 3168017B, 0541706
145Nd CD138, 1, DL-101, Fluidigm, 3145003B, 2651706
159Tb CD161, 1, HP-3G10, Fluidigm, 3159004B, 1361705
156Gd CD183 (CXCR3), 1, G025H7, Fluidigm, 3156004B, 2771708
175Lu CD194 (CCR4)#, 1, 205410, Fluidigm, 3175021A, 2791705
141Pr CD196 (CCR6), 1, 11A9, Fluidigm, 3141014A, 0751705
163Dy CD294 (CRTH2), 1, BM16, Fluidigm, 3163003B, 1671717 & 691605
170Er HLA-DR, 1, L243, Fluidigm, 3170013B, 3571502
161Dy CD152 (CTLA4), 1, 14D3, Fluidigm, 3161004B, 2651711
169Tm CD30*, 1, 81337, R&D, MAB229, Not available
158Gd CD11c*, 1, 3.9, Biolegend, 301639, Not available
173Yb FceR1/ FCER1A*, 1, AER-37 (CRA1), Thermo Fisher Scientific, 16-5899-82, Not available
176Yb TCRγδ*, 1, B1.1, Thermo Fisher Scientific, 16-9959-81, Not available

Notes, * in-house conjugation using Maxpar X8 Antibody Labeling Kits. # this antibody was discontinued. The provider Fluidigm is now switched to Standard Bio.

List 2 (Supplemental Table 3). The list of other CyTOF kits used in this work:
Kit Component, Manufacturer, Catalogue#
Maxpar Thulium Chloride 169Tm—50 mM, Fluidigm, 201169A
Maxpar Gadolinium Chloride 158Gd—50 mM, Fluidigm, 201158A
Maxpar Ytterbium Chloride 173Yb—50 mM, Fluidigm, 201173A
Maxpar Ytterbium Chloride 176Yb—50 mM, Fluidigm, 201176A

List 3 (Supplemental Table 4). The list of flow cytometry Abs used to sort Th2 cells from each participant at each time point:
Target, Alternative name, Fluorochrome, Clone, Used Ab volume ul/1E6 cells, Catalogue#, Manufacturer
CD4 BUV805, SK3, 4, 564910, BD
CD183 (CXCR3) Bv421, 1C6/CXCR3, 3, 562558, BD
CD45RA Pacific Blue, HI100, 2, 304123, Biolegend
CD3 BV510, HIT3a, 3, 564713, BD
CD8 Bv650 ,RPA-T8, 1, 301042, Biolegend
CD127 (IL-7R) Bv711, A019D5, 4, 351328, Biolegend
CD25 (IL-2RA) BB515, 2A3, 3, 564467, BD

List 4 (Other flow cytometry Abs we used for Breg CpG stimulation experiment):
Target, Fluorochrome, Clone, Catalogue#, Manufacturer, dilution factor
CD3 BUV737, HIT3a, 741822, BD, 1/200
CD19 PercP-Cy5.5, HIB19, 45-0199-42, Invitrogen/Thermo Fisher Scientific, 1/200
IL-10 APC, JES3-19F1, 506807, Biolegend, 1/50
TNF BV650, MAb11, 502938, Biolegend, 1/50

Validation	Reaction species and applications of all the primary Abs used in this study have been validated by the given manufactures, as directly stated in the specific datasheet of the given abs. Furthermore, all the Abs were purchased from leading reliable manufactures (BD, Biolegend, or Fluidigm/Standardbio), the general routine validation statements for flow cytometry or mass cytometry analysis are also available on the website of the corresponding provider. For BD, please visit: https://www.bdbiosciences.com/en-lu/products/reagents/flow-cytometry-reagents/research-reagents/quality-and-reproducibility; For Biolegend, please visit: https://www.biolegend.com/en-us/quality/quality-control.<br><br>We have also obtained specificity and quality certificate for each used CyTOF abs from the provider (Standard Bio, previously called as Fluidigm).<br><br>For the validation information of the specific Thermo Fisher Scientific Abs tested with the cytometry application, please visit https://www.thermofisher.com/antibody/product/FceR1-alpha-Antibody-clone-AER-37-CRA1-Monoclonal/16-5899-82; https://www.thermofisher.com/antibody/product/TCR-gamma-delta-Antibody-clone-B1-1-Monoclonal/16-9959-81; https://www.thermofisher.com/antibody/product/CD19-Antibody-clone-HIB19-Monoclonal/45-0199-42<br><br>To review the validation information of the specific R&D Ab tested with the cytometry application, please visit https://www.rndsystems.com/products/human-cd30-tnfrsf8-antibody-81337_mab229

Clinical data

Policy information about clinical studies

All manuscripts should comply with the ICMJEguidelines for publication of clinical research and a completedCONSORT checklist must be included with all submissions.

Clinical trial registration	The study was approved by the Luxembourg National Research Ethics Committee (CNER) (SYS-T-Act: No. 201509/11) and preregistered at ClinicalTrials.gov (NCT02931955) (https://clinicaltrials.gov/study/NCT02931955) on Oct 2016.
Study protocol	The full study protocol is provided with the original manuscript submission and the final submission, and the detailed protocol has been also provided (although split into different sections on the webpage) at ClinicalTrial.gov (https://clinicaltrials.gov/study/NCT02931955) when we registered the study.
Data collection	The study participants with moderate-to-severe allergic rhinitis (pollen allergy patients, PAP, n=16) and insect sting reactions (venom allergy patients, VAP, n=18) eligible for AIT in accordance with the current international guidelines were recruited at a tertiary center (Service National d'Immuno-Allergology of Centre Hospitalier de Luxembourg) between August 2016 and February 2018. The healthy controls (HC, n=10) were recruited at the Clinical and Epidemiological Investigation Center of the Luxembourg Institute of Health (CIEC-LIH) between Jan and Apr 2018.<br><br>In VAP, an ultra-rush AIT protocol was administered to reach the maintenance dose within 8 hours with 14 subcutaneous injections (Fig. 1a). For PAP, a conventional up-dosing AIT was applied to reach the maintenance dose within 6 weeks with a weekly subcutaneous injection scheme. As no comparable ultra-rush up-dosing AIT products were licensed for pollen allergy, neither at the start of the project nor subsequently, the exact sampling periods differed between VAP and PAP. Thus, our sampling schemes were critically chosen to cover comparable phases during the build-up of AIT until the targeted maintenance dose of allergens was achieved for both types of AIT. We performed time-series sampling of peripheral blood during the first seven days, including baseline before AIT, in VAP. As for PAP, we extended our sampling period to two weeks (2W), six weeks (6W) and 12 weeks (12W). To benchmark with the natural immune fluctuations, we also recruited 10 healthy controls (HC), who did not receive any AIT or other immunotherapy, and sampled seven times within 12 weeks to match various time points of both patient groups.<br><br>On the first day of the ultra-rush protocol, most of the VAP received their first injection at 8:00 AM with a gradually increasing dose in µg (i.e., starting from 0.003 to 0.006, 0.015, 0.030, 0.060, 0.150, 0.30, 0.60, 1.50, 3.00, 6.00, 15.00, 30.00, ending with 55.00) at 15-min intervals. While the baseline (0h) blood sample was drawn at 8:00 AM, the second blood sample (8h) was taken at 16:00. There were a few exceptions, where the first injection was given at 8:15 AM and then the afternoon sampling was delayed to 16:15. Biological samples were collected by trained study nurses, transported at room temperature (RT) within maximum two hours to the bio-specimen laboratory of the Integrated Biobank of Luxembourg (IBBL), where they were either used freshly in the experiments on the same day, or cryopreserved in the IBBL biorepository for further analysis later on.<br><br>17 out of 18 VAP have completed the full 3-5-year AIT (Supplementary Table 1) except for one patient who stopped around half a year later due to the comorbidity with rheumatoid arthritis. 14 of 18 VAP have collected at least one long-term (>6 Months) follow-up plasma sample (for details, please refer to Source Data). The curative outcome was essentially evaluated based on the reaction to the reported field sting(s). Although skin tests with insect venom extract are not regarded as a reliable marker to distinguish cured tolerant individuals from patients with a relapse, it is generally agreed that a negative venom skin test could be used as a good surrogate marker of clinical cure in the vast majority of cases. Thus, we also analyzed skin test negativity at AIT termination.<br><br>Only one PAP stopped the AIT approximately two years after the onset for personal reasons (moving to another country) while everyone else in the PAP group has finished the full 3-5-year AIT course. 14 out of 15 assayed PAP have provided at least one long-term follow-up plasma sample (for details, please refer to Source Data). The allergy specialists from Centre Hospitalier de Luxembourg retrospectively evaluated the outcomes of pollen AIT for each patient after each pollen season during the 3-5 years mainly by applying the principles of the Combined Symptom Medication Score (CSMS) for the peak of the last pollen season before inclusion and the last pollen season under pollen AIT (Supplementary Table 1).
Outcomes	The primary outcome of this observational study is the comprehensive immunological cellular characterization during AIT initiation phase, whereas the secondary outcomes are the serological and Th2-cell-type-specific transcriptomic analyses.

Plants

Seed stocks	N.A., as we did not use plants in this study.
Novel plant genotypes	N.A., as we did not use plants in this study.
Authentication	N.A., as we did not use plants in this study.

Flow Cytometry

Plots

Confirm that:

- ☒ The axis labels state the marker and fluorochrome used (e.g. CD4-FITC).
- ☒ The axis scales are clearly visible. Include numbers along axes only for bottom left plot of group (a 'group' is an analysis of identical markers).
- ☒ All plots are contour plots with outliers or pseudocolor plots.
- ☒ A numerical value for number of cells or percentage (with statistics) is provided.

Methodology

Sample preparation

PBMCs (peripheral blood mononuclear cells) were obtained from fresh whole blood by density gradient immediately followed by CD4 T-cell isolation and Th2 sorting or cryopreservation. For PBMC isolation and deep immunophenotyping analysis, we followed a similar procedure as we described in another clinical study (Capelle et al. 2022). Briefly, we collected up to 50 ml of blood per patient at each time point. 40 ml were collected in BD ACD tubes for PBMC analyses and 10 ml in BD EDTA tubes for whole-blood-count and cytokine measurements. We first added 13 ml of Ficoll Paque Plus (GE17-1440-02, Merck) at the bottom of the Falcon tubes. Blood from ACD tubes was mixed and split into two 50-ml Falcon tubes that were diluted with up to a total volume of 35 ml with DPBS (14190144, Thermo Fisher Scientific). Diluted blood was added carefully on the top of Ficoll-containing Falcon tubes. The Falcon tubes were then centrifuged at 400g at room temperature (RT) for 30 min. The PBMC layer was collected, diluted with DPBS plus 2% heat-inactivated FBS (fetal bovine serum, 10500-064, Thermo Fisher Scientific) and centrifuged twice at 300g for 10 min at RT. Purified PBMCs were either cryopreserved using 90% heat-inactivated FBS plus 10%DMSO (D2650, Sigma Aldrich) freezing medium and stored in liquid nitrogen or used freshly to isolate CD4+ T cells as described below. The plasma layer was also collected during the PBMC isolation process. Whole-blood-count data were measured by ABX Micros CRP 200 (Horiba, Axonlab) and plasma were isolated from fresh blood collected in 10 ml BD EDTA tubes.

Peripheral blood mononuclear cells (PBMCs) were isolated and stored in liquid nitrogen until the day of the staining when the cryovials were thawed using warm DMEM 4.5 g/L Glucose with L-Glutamine (BE12-604F, Lonza) containing 5% of FBS (10500-064, Thermo Fischer Scientific) and 2 mM EDTA (15575-038, Invitrogen). Cells were then washed with PBS (17-516F, Lonza). Prior to the metal-conjugated antibody staining and for assessing their viability, cells were re-suspended at a concentration of 1E7 / ml and were incubated for 5 min at RT with Cell-ID Cisplatin (201064, Fluidigm) at a final concentration of 1 µM. The incubation was stopped by adding the staining buffer (PBS, 5%FBS, 2 mM EDTA; of note, EDTA was not used in other scenarios unless specified). Surface staining was performed by adding a cocktail of pre- or in-house-conjugated antibodies (Supplementary Table 2) for 30 min at RT; excess antibodies were removed by washing (400g, RT, 10 min). It is important to mention that each tube was then split into half for introducing intense washing steps since the samples had initially shown strong signals of iodine (most probably coming from the Ficoll). As a last step, samples were incubated with Ir-Intercalator (201192B, Fluidigm), diluted in MaxPar Fix&Perm (201067, Fluidigm) at a final concentration of 50 nM, and rested at 4oC until the day of the acquisition. Prior to the acquisition, cells were washed twice with PBS and with de-ionized water two times. Centrifugation conditions after fixation, on the acquisition day, were 800g, for 10 min at 4oC. Cells were re-suspended at 5E5 per ml in 1:10 calibration beads (EQ Four Element Calibration Beads, 201078, Fluidigm), diluted with de-ionized water and the samples were analyzed with the Helios mass cytometer (Fluidigm) at a flow rate of 0.030 ml per min.

For Breg stimulation analysis, following the thawing of bio-banked cryopreserved PBMC depleted of CD4 T cells [refer to the section 'Isolation and cryopreservation of PBMCs'], we re-suspended those cells in RPMI complete media [RPMI-1640 (Lonza, 12167F) supplemented with 100 U/ml Penicillin plus 100 µg/ml Streptomycin (Gibco, 15140-122), 2 mM L-Glutamine (Gibco, A2916801) and 10% heat-inactivated FBS (Gibco 10500064)]. 8.5 x 105 cells were stimulated by 1 µM of CpG (Invivogen, ODN2395) for 72h in 200 µl of RPMI complete media with a U-bottom 96-well plate. In the last 4.5h before staining, 50 ng/ml PMA (Sigma Aldrich, 16561-29-8), 250 ng/ml Ionomycin (Sigma Aldrich, 13909-1ML) plus 1 µl of Golgiplug Protein Transport Inhibitor (BD, 555029) were added together. The cells were then washed (200g, 10 min, 4oC) and blocked with NA/LE Fc blocker (BD, 564765) for 5 min at 4oC. The cells were again washed and stained with the surface marker antibody mastermix (CD3 BUV737, 741822, BD, dilution 1:200; CD19 PercP-Cy5.5, 45-0199-42, Invitrogen, dilution 1:200; LIVE/DEAD Fixable Near-IR Dead Cell Stain, L10119, Thermo Fisher Scientific, dilution 1:500) in BD Brilliant stain buffer at 4oC for 30 min. The cells were subsequently stained with selected cytokine antibodies (IL-10-APC, 506807, Biolegend, dilution 1:50; TNF-BV650,

	502938, Biolegend, dilution 1:50) using the Fixation/Permeablization kit (554714, BD) following the manufacture's recommendations. The cells were finally re-suspended in the staining buffer (2% FBS in Ca2+/Mg2+ free PBS) and acquired in a BD LSRFortessa analyzer (v8.0.1).
Instrument	For FACS sorting, we used BD Aria III sorter. For CyTOF analysis, the Helios system (Fluidigm) was used.
Software	For CyTOF acquisition software: CyTOF Helios software v7.0;<br>The sorting acquisition software: FACSDiva v8.0.1;<br>The analysis software for most of cytometry experiments was FlowJO V10 while for Breg stimulation measurement, the results were analyzed in Kaluza software (v2.1, Beckman).
Cell population abundance	The relative population abundance of each analyze subset has been quantified by CyTOF or FACS Sorting following the provided gate strategies. The detailed frequency information (mean and deviation) has been provided through different main and Supplementary Figures. Importantly, through our interactive i3Dare website, for each immune subset, we provide the quantitative information for each individual participant at the each time point.<br><br>The purity of the sorted targeted T-cell subsets has been checked in the setting development experiments. Following the successful setting experiments, we applied the same application setting to all the following experiments. Due to a very limited number of sorted target rare subset that was strictly defined by more than 10 different cell surface markers (the obtained cell number was often lower than 100K for Th2 cells), we did not perform post-sorting purity checking for each sorting experiment to save the precious patient cells. Furthermore, the high Th2 purity was confirmed by the high expression of the known marker genes of CD4 Th2 cells, but not of Th1, Th17 and Treg subsets using our cell-type-specific RNA-seq dataset generated from the sorted Th2 cells.
Gating strategy	We have already provided the gating strategy examples in Figure 3, Figure 4 and Supplementary Figure 1, 3, 4 and 6. The complete gating strategy for different immune subsets by CyTOF analysis was provided in Supplementary Figure 1. In the meantime, we also provided the detailed analysis strategy in our interactive website i3Dare (refer to Methods).

☒ Tick this box to confirm that a figure exemplifying the gating strategy is provided in the Supplementary Information.
